# An inositol receptor Itr4 coordinates carbon catabolite regulation and virulence in *Cryptococcus neoformans*

**DOI:** 10.1371/journal.pbio.3003709

**Published:** 2026-10-13

**Authors:** Yina Wang, Robert J. Tancer, Maggie Wear, Katrina M. Jackson, Yu Zhang, Kan Li, Jun Wang, Yong-Gui Gao, Kirsten Nielsen, Arturo Casadevall, Chaoyang Xue

**Affiliations:** 1 Public Health Research Institute, New Jersey Medical School, Rutgers University, Newark, New Jersey, United States of America; 2 Department of Microbiology, Biochemistry and Molecular Genetics, New Jersey Medical School, Rutgers University, Newark, New Jersey, United States of America; 3 Department of Molecular Microbiology and Immunology, Johns Hopkins Bloomberg School of Medicine, Baltimore, Maryland, United States of America; 4 Pathogen and Microbiome Institute, Northern Arizona University, Flagstaff, Arizona, United States of America; 5 Department of Medicinal Chemistry, Ernest Mario School of Pharmacy, Rutgers University, Piscataway, New Jersey, United States of America; 6 School of Biological Sciences, Nanyang Technological University, Singapore, Singapore; 7 Department of Biomedical Sciences and Pathobiology, College of Veterinary Medicine, Virginia Tech, Blacksburg, Virginia, United States of America; University of Birmingham, UNITED KINGDOM OF GREAT BRITAIN AND NORTHERN IRELAND

## Abstract

Nutrient sensing and utilization are critical for microbes to adapt to their environment and evade predators. The encapsuled yeast *Cryptococcus neoformans* has a unique ability to sense and utilize the sugar inositol as both a signaling molecule and as a carbon source. This trait is advantageous not only in the natural environment but also to promote its pathogenesis, including invasion of the central nervous system. In this study, we have identified a novel inositol transporter-like receptor (transceptor) Itr4 that coordinates inositol and glucose metabolism by regulating inositol transporters (*ITR*s) and glucose sensor Hxs1. Itr4 binds inositol and regulates the function of other *ITR*s and its loss leads to a significant decrease in inositol uptake activity, resulting in a lack of mating, reduced capsule size, reduced blood–brain barrier (BBB) penetration, and virulence attenuation. Mutagenesis analysis of Itr4 protein identified an essential N-terminal tail and two amino acids, Q388 and Q389, as sites of functional importance. The *ITR4*^*Q388A, Q389A*^ allele showed a dominant phenotype with increased inositol uptake, enlarged capsule and significantly attenuated virulence. In summary, we identify a new *C. neoformans* inositol transceptor, Itr4, the first such example in eukaryotes, with novel regulatory roles in both inositol and glucose metabolism and fungal virulence.

## Abstract 2

How pathogens prioritize nutrients while adapting to changing environments is central to their survival and ability to cause disease. This study shows that the inositol-sensing receptor Itr4 enables Cryptococcus neoformans to maintain inositol use even when glucose is abundant, linking nutrient sensing to carbon metabolism and virulence.

## Introduction

The human fungal pathogen *Cryptococcus neoformans* is the leading cause of life-threatening fungal meningoencephalitis that accounts for ~15% of AIDS-related deaths annually [1,2]. Cryptococcal meningitis occurs after aerosolized fungal cells or spores dispersed in the environment are inhaled and disseminate from the lung to the brain via the bloodstream [3,4]. The medical significance and genetic tractability of *C. neoformans* have propelled extensive studies on the mechanism of its virulence with significant progress in identifying factors required for fungal pathogenesis [5–8]. However, our understanding of the molecular basis of cryptococcal pathogenesis, including meningoencephalitis remains incomplete.

In previous studies, we have shown the importance of fungal myo-inositol acquisition and metabolism in *Cryptococcus* species [9]. Interestingly, environmental reservoirs for *Cryptococcus*, such as *Eucalyptus* spp., which is also important for cryptococcal mating, as well as the natural fungal predator amoeba both contain high levels of inositol [9,10]. In addition to environmental niches, inositol is highly abundant in the brain, promotes fungal traversal of the blood–brain barrier (BBB) and plays a critical role in host–pathogen interactions during infection of the central nervous system (CNS) [11–13]. The cryptococcal genome also reflects the evolutionary adaptations associated with the expanded role of inositol in this organism [9,11]. Therefore, we reasoned that an evolutionary specialization that may have originally promoted survival on natural hosts also facilitates the infection of the human host.

Fungal cells acquire inositol through two mechanisms. One is the internal synthesis of inositol by converting intracellular glucose into inositol in a multiple-step biosynthetic pathway [14,15]. Inositol can also be imported from the extracellular environment via inositol transporters [16–20]. The inositol transporter (*ITR*) gene family is part of the sugar transporter superfamily and plays an important role in inositol acquisition in fungi. Our previous studies showed that *C. neoformans* contains an expanded *ITR* gene family in its genome [11]. The *C. neoformans ITR* homologs can be divided into two distinct subgroups (I and II). The seven members of subgroup I (*ITR1, ITR1A, ITR2, ITR3, ITR3A, ITR3B,* and *ITR3C*) share high sequence homology with well-characterized *ITR*s in other fungi, such as *S. cerevisiae* and *C. albicans*. Subgroup II has three members (*ITR4, ITR5,* and *ITR6*) that are more distantly related *ITR*s that are more closely related to *HGT19*, a potential second *ITR* in *C. albicans* [21]*.* The expansion of this gene family suggests an important biological significance for inositol sensing, transporting and utilization in this fungus. Our previous functional studies on the seven group I *ITR*s have revealed that Itr1A and Itr3C are major low-affinity inositol transporters required for fungal sexual reproduction and fungal virulence in murine infection models [21]. The *itr1a*Δ *itr3c*Δ double mutant has a virulence defect, with a reduced ability to penetrate across the BBB that leads to virulence attenuation during brain infection [13]. This result suggests that inositol acquisition is important for the pathogenicity of *C. neoformans*. Because fungal *ITR*s are proton-dependent importers, distinct pharmacokinetically from sodium-dependent human and animal myo-inositol transporters (MITS) [20], fungal *ITR*s may be developed as a valuable drug target.

Genome-wide association studies (GWAS) conducted on clinical isolates collected during the Cryptococcal Optimal Antiretroviral Therapy Timing (COAT) Trial in Uganda [22], also identified *ITR4,* a member of subgroup II*,* as one of several genes having polymorphisms associated with differential human immune responses and patient survival [23,24], suggesting the clinical importance of inositol utilization. However, the mechanisms for how fungal cells sense, take up and utilize intracellular inositol to influence its development and virulence remain poorly understood. The function of subgroup II *ITR*s has not been characterized.

In this study, we analyzed the function of the group II *ITR* genes in *C. neoformans* and found that Itr4 is the only one with significant impact on mating and fungal virulence. Using a *S. cerevisiae* heterologous expression system, we confirmed that Itr4 functions as an inositol sensor rather than a transporter. Mutagenesis studies showed that Itr4 had an outsized role in regulating fungal inositol uptake and fungal virulence in a murine model of systemic cryptococcosis. We further identified the potential inositol-binding sites in Itr4 and generated *ITR4*^*Q388A, Q389A*^ mutant allele based on site mutagenesis. Significantly, although the *itr4*Δ mutant produced a smaller capsule while the *ITR4*^*Q388A, Q389A*^ allele produced an enlarged capsule, both mutants showed significant virulence attenuation in murine infection models. These results support the conclusion that inositol sensing, uptake and utilization in *C. neoformans* is important for the *Cryptococcus*-host interaction, BBB crossing and fungal virulence.

## Results

### Itr4 is a group II *ITR* homolog required for polysaccharide capsule formation

The production of a polysaccharide capsule is a key virulence factor that plays a critical role in *Cryptococcus* pathogenesis. Previously, we demonstrated that inositol regulates both capsule size and polysaccharide structure [21,25]. We also identified the expanded gene family of inositol transporters (*ITR*s) and inositol oxygenases (*MIO*s) and revealed that both the two major group I *ITR*s (*ITR1A* and *ITR3C*) and *MIO*s are required for fungal virulence [25]. However, the function of the group II *ITR*s (*ITR4, ITR5* and *ITR6*) has not been studied in detail. To further understand the role of the group II *ITR*s in capsule regulation, we generated the *itr4∆*, *itr5∆*, and *itr6∆* single mutants and their triple mutants (*itr4∆ itr5∆ itr6∆*) and measured the size of their capsules. We found that while the *itr5∆* and *itr6∆* single mutants did not exhibit obvious capsule difference, both the *itr4∆* single mutant and the triple mutant cells had significantly reduced capsule and cell size (Fig 1A and 1B). The capsule defect in *itr4∆* was fully complemented by reintroducing the *ITR4* allele. In addition to having a capsule, thermotolerance and melanin production are two other classical virulence factors in *C. neoformans*. Our phenotypic analysis showed that all three *itr* single and triple mutants had normal growth at 37 °C with proper melanin production (S1A and S1B Fig).

**Fig 1:**
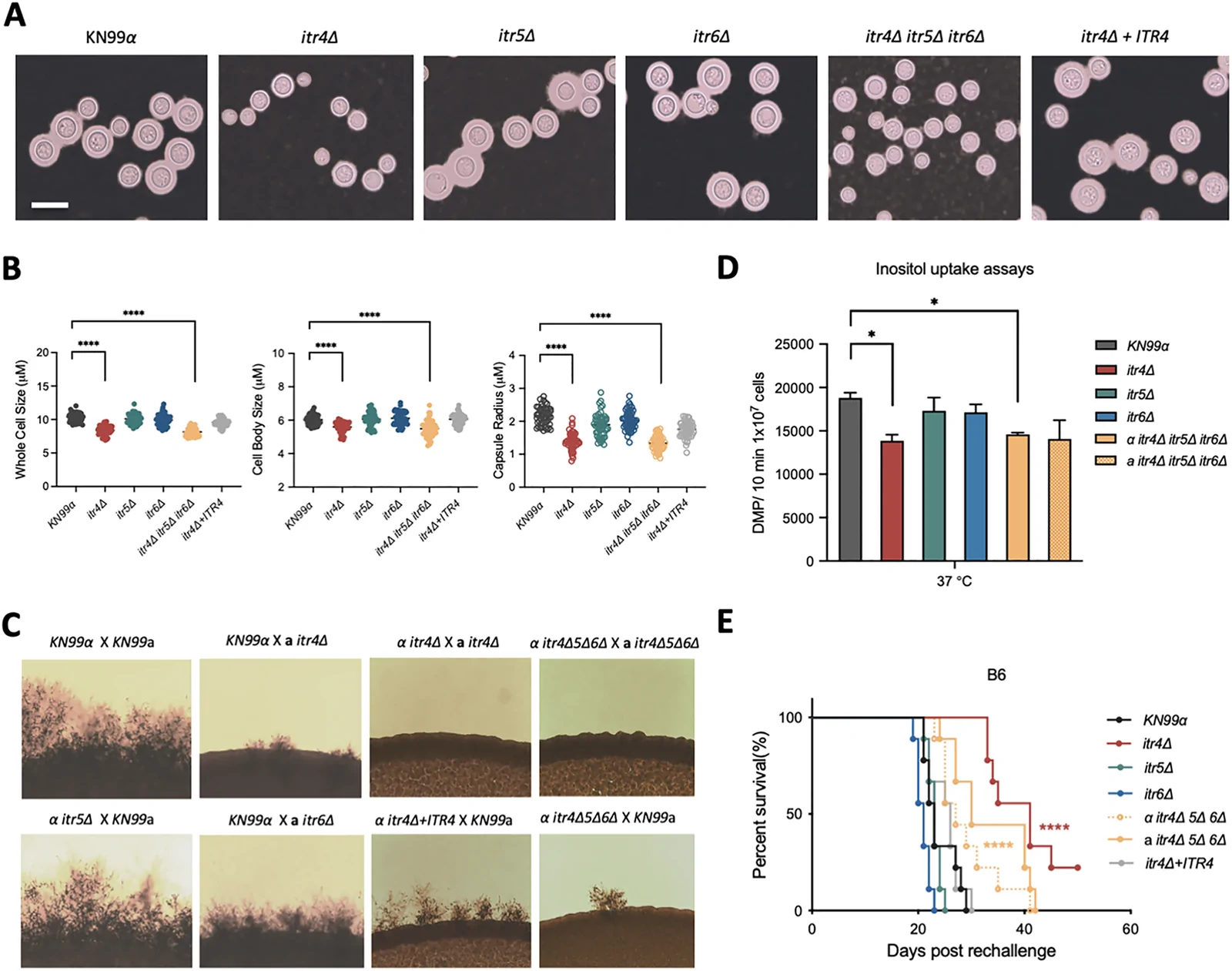
Analysis of group II *ITRs* through in vitro phenotype, in vivo virulence and inositol uptake. **(A)** Cell morphology of KN99α, *itr4∆, itr5∆,* and *itr6∆* single mutants and their triple mutants (*itr4∆ itr5∆ itr6∆*) after grown on DME medium for 3 days. **(B)** Quantitative data of both cell size and capsule size from over 50 cells from each sample are presented. Statistical analysis was done using two-tailed Student *t* test. “****”, *P* < 0.0001. Bar, 10 µm. **(C)** Unilateral and bilateral mating assays were performed in MS medium. Mating cultures were incubated at room temperature in the dark for 10 days before mating hyphae were photographed. **(D)** Inositol uptake analysis of *C. neoformans* strains. Yeast cells were mixed with 3^H^-labeled inositol and incubated at 30 °C for 10 min in triplicate (repeated three times with similar patterns). Error bars indicate standard deviations of results from the three replicates. **(E)** Survival rates of mice infected with KN99α, *itr4∆, itr5∆,* and *itr6∆* single mutants and their triple mutants *itr4∆ itr5∆ itr6* in a murine intranasal inhalation model. Groups of 10 mice were infected with 1 × 10^4^ yeast cells each strain. For the animal survival data: “****”, *P* < 0.0001. Statistical analysis was done using a log-rank (Mantel-Cox) test. The data underlying this figure can be found in the supplemental information file S1 Data.

We also evaluated the possibility that group II *ITR*s could be involved in stress responses. In our in vitro phenotypic assays, the *itr* null mutants showed normal growth under osmotic stress (1.5 M NaCl or 1.0 M Sorbitol) and oxidative stress (3.0 mM H_2_O_2_) (S1C Fig). We further examined the cell integrity of *itr* mutants by applying chemicals that target cell integrity, including SDS, calcofluor white (CFW), and Congo Red. SDS disrupts the plasma membrane and lyses cells with a defective membrane, while CFW specifically binds to chitin and Congo Red binds to glucan and chitin in the cell wall to disrupt cell wall integrity. Our results showed that the *itr4∆* mutant had a growth defect on SDS but not CFW or Congo Red, indicating a defect in plasma membrane integrity (S1D Fig).

### Itr4 regulates sexual reproduction

We previously found that inositol is important for stimulating *Cryptococcus* mating to help the fungus complete its life cycle and several *ITR*s in Group I are required for fungal mating [11,26]. To determine the potential involvement of group II *ITR*s in fungal mating, we tested their single and triple mutants in both unilateral (mutant × wild type) and bilateral (mutant × mutant) mating assays on MS medium. Significant reduction of mating hyphal production and defects in sporulation were observed in the unilateral mating assays of the *itr4∆* single mutant and *itr4∆ itr5∆ itr6∆* triple mutant, while the mating was completely blocked in their bilateral mating assays. No obvious mating defect was observed in unilateral and bilateral mating assays of *itr5∆* and *itr6∆* single mutants. The mating defect of *itr4∆* could be fully complemented (Fig 1C), demonstrating that Itr4 is essential for fungal mating.

### Itr4 regulates inositol uptake activity

To understand whether the mating and capsule formation defect of the *itr* mutants were related to the defect in inositol uptake activity, we performed inositol uptake assays for parental wild type KN99α and the group II *itr* single mutants, as well as the *itr4∆ itr5∆ itr6∆* triple mutant. Consistent with our previous report [21,23], our results showed that the *itr4∆* mutant had significantly reduced inositol uptake activity when compared to KN99α*.* Similar to *itr4∆,* the triple mutants also manifested reduced inositol uptake activity, while *itr5∆* and *itr6∆* single mutants showed wild-type level of inositol uptake activity (Fig 1D). These results also indicate that Itr4 is the main *ITR* in group II with important biological function.

### Itr4, but not Itr5 or Itr6, is required for full fungal virulence

Our previous studies showed that Itr4 is required for full cryptococcal virulence, and the outcome of infection in animal models is mouse strain-dependent, due to difference in host immune responses [23,24,27]. Here we evaluated the potential involvement of every group II *ITR* in fungal virulence in a murine infection model of systemic cryptococcosis. Ten C57BL/6 mice per group were inoculated with 1 × 10^4^ yeast cells per mouse of each strain intranasally, and their survival rates were determined. Our results showed that while mice infected with the *itr4∆* mutant, or the *itr4∆ itr5∆ itr6∆* triple mutant had delayed mortality, the ones infected with the *itr5∆* or *it6∆* mutants showed a survival rate similar to KN99α-infected ones. The virulence attenuation exhibited in *itr4*∆ was fully rescued by reintroducing the *ITR4* allele (*itr4∆* + *ITR4*) in the same murine model (Fig 1E). These results demonstrate that Itr4, but not Itr5 or Itr6, was required for full virulence, which is consistent with our previous report that Itr4 is required for full fungal virulence in C57BL/6 mice [23]. Our previous studies also demonstrated that the *ITR4* gene showed a significant polymorphism in clinical isolates from patients with cryptococcal meningitis [23]. Therefore, we focused on the functional analysis of Itr4 to better understand the inositol regulation of *Cryptococcus* pathogenesis.

### Itr4 is an inositol transceptor regulating inositol sensing

To better characterize Itr4 function, we expressed *ITR4* in a *Saccharomyces* mutant strain with deletions of both *ITR1* and *ITR2* (*Sc itr1 itr2*) that lacks inositol uptake activity [21]. Full-length cDNAs of *ITR4* was cloned into the yeast expression vector pTH74 under the control of the *ADH1* promoter to generate a *ITR4:GFP* fusion construct. The strains expressing *ITR4:GFP* localized most of their GFP fluorescence signals to the plasma membrane. The control strain expressing the empty vector had diffuse GFP fluorescence signal in cytosol (Fig 2A).

**Fig 2:**
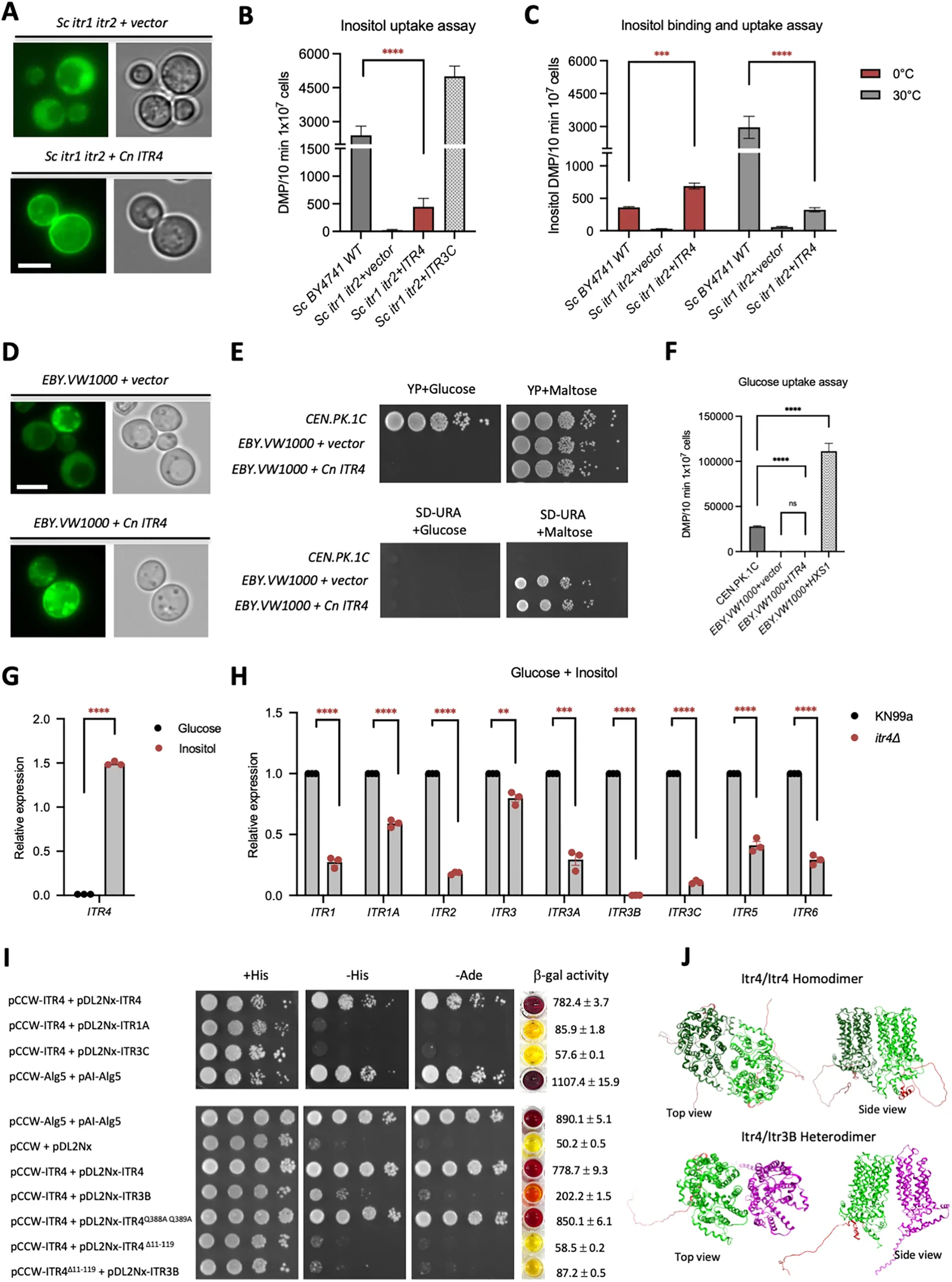
Heterologous expression and uptake assays of *ITR4* in *Saccharomyces* strains lacking inositol transporter. **(A)** Localization of *Itr4* in *S. cerevisiae* was determined by overexpressing a *Itr4:GFP* fusion protein in *Sc itr1 itr2*, a mutant lacking two inositol transporters. **(B)** Inositol uptake analysis of *Cryptococcus ITR* genes expressed in a yeast heterologous system was performed by detecting 3^H^-labeled inositol signal after 10 min incubation at 30 °C. Error bars indicate standard deviations of data from at least three independent experiments. Statistical significance was determined using an unpaired two-tailed Student *t* test. “****”, *P* < 0.0001. *Sc*, *S*. *cerevisiae*. **(C)** Inositol binding assay. Yeast cells were mixed with 3^H^-labeled inositol and incubated at 0 °C for 10 min. At least three independent biological replicates were performed. Error bars indicate standard deviations of data from independent experiments. Statistical significance was determined using an unpaired two-tailed Student *t t*est. “****”, *P* < 0.0001, “***”, *P* < 0.001 **(D)** Localization of Itr4 in *S. cerevisiae* was determined by overexpressing a *Itr4:GFP* fusion protein in EBY.VW1000. EBY.VW1000 expressing an empty vector was used as a control. **(E)** Growth assay of *Saccharomyces* strains expressing *ITR4* on plates with indicated carbon sources. Cultures of the background strain CEN.PK2.1C and EBY.VW1000 expressing the pTH74 empty vector, or the *ITR4* gene were inoculated on SD media lacking Uracil but containing 2% maltose as carbon source. Serial 10-fold dilutions were prepared, and 5 µl of each dilution was spotted on different plates and incubated at 30 or 37 °C for 48 h before photography. **(F)** Glucose uptake assay was performed for CEN.PK2.1C and EBY.VW1000 expressing the empty vector, *HXS1* or *ITR4*. Yeast cells were mixed with 3^H^-labeled glucose and incubated at 30 °C 10 min. The error bar indicates the standard deviation of at least three repeats. Statistical significance was determined using an unpaired two-tailed Student *t* test. “****”, *P* < 0.0001; ns, no*t* significant. **(G)** Quantification of the *ITR4* expression in a qRT-PCR assay. RNAs were prepared from KN99**a** cells cultured on medium with YNB + 1% glucose or YNB + 1% inositol for 4 **h.** Values are expressed as relative expression of the *ITR4* gene, normalized to the *GAPDH* gene endogenous reference. **(H)** The expression of *ITRs* under KN99**a** and *itr4∆* mutant strain background was determined in a qRT-PCR assay. The data underlying this panel can be found in the supplemental information file S1 Data. **(I)** Detection of potential interactions between Itr4 and Itr1A, Itr3C, Itr3B, Itr4^∆11-119^ and Itr4^Q388A, Q389A^ in a yeast split-ubiquitin system. Two constructs, pCCW-Alg5 and pAI-Alg5, and pCCW and pDL2Nx serve as positive and negative controls. Interaction was determined by the growth of yeast transformants on medium lacking histidine or adenine, and also by measuring β-galactosidase enzyme activities to verify the interaction. **(J)** Alphafold 2 protein modeling of Itr4-Itr4 homodimer and Itr4-Itr3B heterodimer structures.

We measured inositol uptake activity for the *Sc itr1 itr2* mutant strain expressing *C. neoformans ITR4* by using ^3^H-labeled inositol (myo-[2-^3^H] inositol) at 30 °C as described previously [21]. The yeast strain expressing *ITR4* had detectable signal but was significantly lower than the parental wild type BY4741 or the *Sc itr1 itr2* strain expressing the major inositol transporter *ITR3C* (Fig 2B), and close to the background noise. To determine whether the weak signal was due to inositol uptake or binding, we performed inositol binding assay at 0 °C and detected a weak ^3^H signal in the *ITR4* yeast-expressing strain that was similar to the signal detected in our uptake assay at 30 °C (Fig 2C). This result showing a lack of inositol uptake in the heterologous system indicates that Itr4 may not function as a typical transporter rather as an inositol sensor (transceptor). Transceptors are membrane proteins that combine transporter and receptor functions by mediating nutrient uptake while simultaneously initiating signaling responses [28].

### Itr4 is not required for glucose uptake activity in a heterologous expression system

There was a possibility that Itr4 may transport other sugars, e.g., glucose. To examine whether Itr4 functions as a glucose transporter, the *ITR4:GFP* fusion construct was expressed under the control of the *ADH1* promoter in the *S. cerevisiae hxt* mutant strain EBY.VW1000, in which all 20 hexose transporters are deleted [29]. The EBY.VW1000 strain thus cannot grow on YPD medium but can grow on maltose as a carbon source. The expression of *ITR4* was confirmed by GFP signals and qRT-PCR. Our results showed that Itr4:GFP was localized on the plasma membrane as expected (Fig 2D).

The *S. cerevisiae* control strain CEN.PK2.1C, EBY.VW1000 expressing the empty vector or *P*_*ADH1*_-*ITR4:GFP* were used for growth assay on YPD or YP+Maltose. On YPD there was no growth of the EBY.VW1000 expressing either empty vector or *ITR4:GFP*, while the growth defect was rescued on YP+Maltose condition, indicating the *ITR4*-expressing strain can’t utilize glucose for their growth, likely due to the lack of glucose uptake by Itr4 (Fig 2E). To further confirm this, we examined the glucose uptake by Itr4 in this heterologous expression strain and found that no glucose uptake was detected in strains expressing *ITR4* or empty vector, while a control strain expressing *Cryptococcus* high-affinity glucose transporter Hxs1 showed glucose uptake activity, confirming that Itr4 is not a glucose transporter (Fig 2F).

We posit that Itr4 may function as an inositol transceptor to regulate inositol uptake and metabolism. Such a sensory function could explain why the *itr4*∆ mutant, in which all other 9 *ITR*s remain present, showed significant defects in inositol uptake, virulence factor development, fungal mating, as well as fungal virulence (Fig 1). To test this hypothesis, we determined the potential role of Itr4 in the regulation of other *ITR* family members by comparing the expression changes of remaining *ITR* genes in KN99**a** and its *itr4*∆ mutant. Our qRT-PCR results showed that the *ITR4* expression level was highly induced in inositol condition compared to glucose condition (Fig 2G), and that most *ITR* genes had reduced expression in *itr4*∆ compared to wild type, indicating that they were upregulated by Itr4 (Fig 2H). Based on this result and also results from Itr4 inositol uptake and binding activity shown in Fig 2C, we confirm that Itr4 is an inositol transceptor.

Receptors often form homodimer or heterodimer structure to sense signals and activate downstream pathways. To test the hypothesis that Itr4 may directly interact with other *ITR*s to form dimer structure to regulate inositol uptake, we performed protein-protein interaction assays using the split-ubiquitin system, a membrane-based yeast two-hybrid system [21]. We detected a strong interaction between Itr4 with itself, a weak interaction with Itr3B, and no interaction with two major inositol transporters Itr1A and Itr3C, based on both yeast growth on SD drop out medium and ß-galactosidase activity (Figs 2I and S2B). These results indicate that Itr4 can form a homodimer and also a heterodimer with Itr3B (Fig 2I). Predicted protein dimer structure models were presented in Fig 2J.

In aggregate, our results demonstrate that Itr4 is an inositol-specific transceptor to sense inositol and regulate inositol uptake in *C. neoformans*. This assessment is consistent with the result that the *itr4*∆ single mutant showed significant defects in inositol uptake and exhibited strong inositol-related phenotypes.

### Itr4 N-terminal tail is required for maintaining the Itr4 membrane localization and function

To better understand the potential domains and amino acid sites required for Itr4 function, we generated a structure model for the Itr4 protein with the AlphaFold 2 program [30] (S3 Fig). Interestingly, protein structural modeling and protein sequence topological analysis showed that Itr4 has a unique long N-terminal cytosolic leader sequence (120 amino acids) before the first transmembrane domain (Figs 3C and S3). Structural modeling suggests that this region may be required for the Itr4 dimerization (S2C Fig). Previous studies reported that glucose transceptors, e.g., Snf3 and Rgt2 in *S. cerevisiae*, have a long C-terminal tail that is required for their function as a glucose sensor by binding to membrane-associated casein kinases [31]. To investigate the potential function of the Itr4 N-terminal leader sequence, we expressed an *ITR4* gene lacking this region (*ITR4*^*∆11-119*^) in the *Sc itr1 itr2* mutant strain (Fig 3A). While the strain expressing *ITR4:GFP* showed plasma membrane localization, the strain expressing *ITR4*^*∆11-119*^ was no longer localized to the plasma membrane and instead showed an exclusively cytosolic GFP signal (Fig 3A). The inositol uptake assay also showed that the *Sc itr1 itr2* strain expressing *ITR4*^*∆11-119*^ did not have uptake activity (Fig 3B).

**Fig 3:**
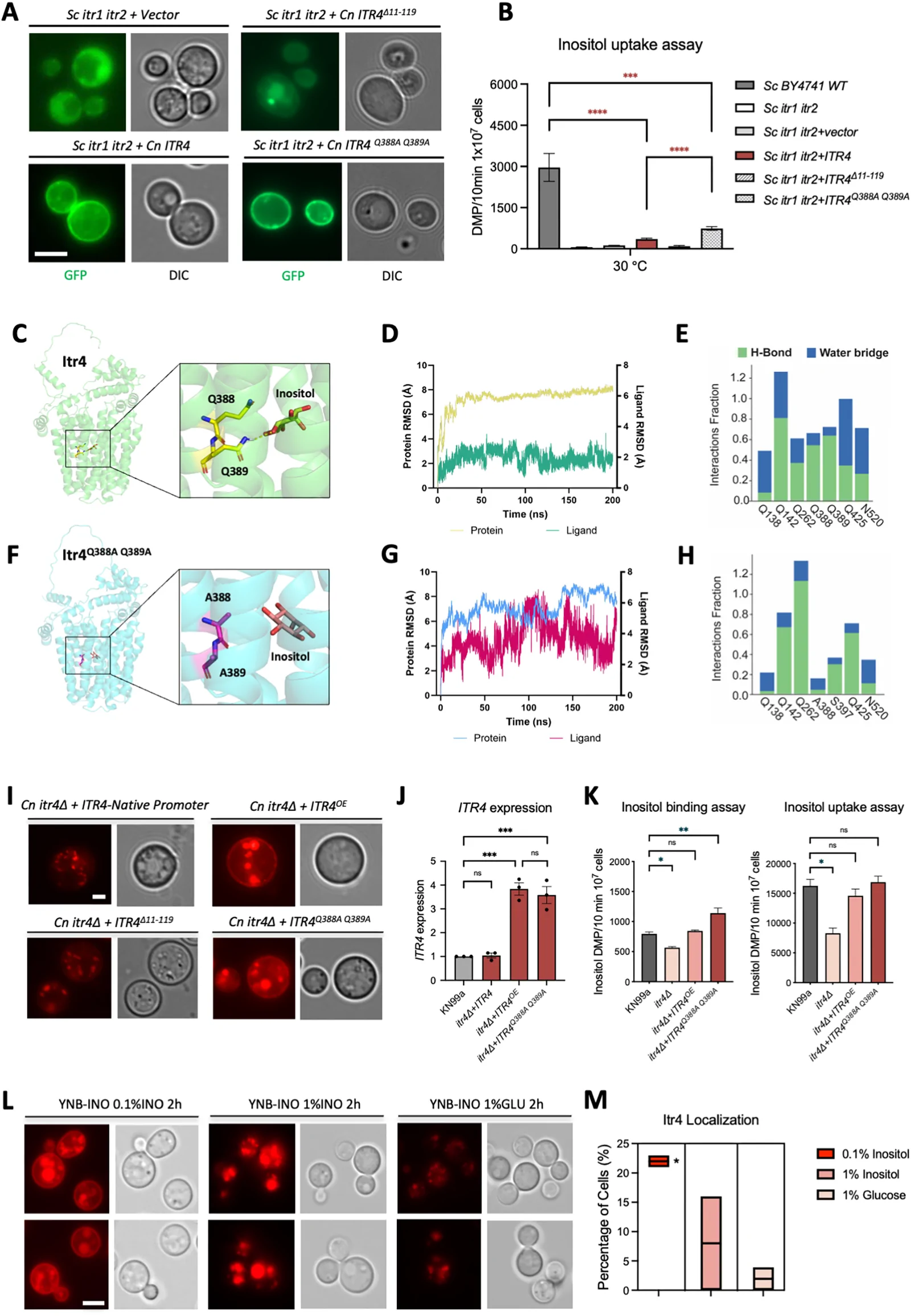
Itr4 protein structure modeling and predicted sugar-binding sites. **(A)** Localization of *ITR4, ITR4*^*∆11-119*^ and *ITR4*^*Q388A, Q389A*^ in *S. cerevisiae* was determined by overexpressing of their GFP fusion protein in *Sc itr1 itr2* mutant lacking two inositol transporters. **(B)** Inositol uptake analysis of *Cryptococcus ITR4, ITR4*^*∆11-119*^ and *ITR4*^*Q388A, Q389A*^ expressed in a yeast heterologous system. Yeast cells were mixed with 3^H^-labeled inositol and incubated at 30 °C for 10 min. Error bars indicate standard deviations of data from at least three independent experiments. *Sc*, *S*. *cerevisiae*. **(C)** Docking pose of inositol in wild-type Itr4. **(D)** Protein and ligand RMSD during a 200-ns MD simulation of the wild-type complex. **(E)** Residue interaction fractions between inositol and wild-type Itr4. **(F)** Docking pose of inositol in the Itr4^Q388A, Q389A^ double mutant. **(G)** Protein and ligand RMSD during a 200-ns MD simulation of the mutant complex. **(H)** Residue interaction fractions between inositol and the mutant transporter. **(I)** Localization of *ITR4, ITR4*^*∆11-119*^ and *ITR4*^*Q388A, Q389A*^ in *C. neoformans* was determined by overexpressing of their mCherry fusion protein in *itr4∆* mutant strain. **(J)** qRT-PCR analysis of *ITR4* gene expression of different promoter. The *GAPDH* gene was used as a reference. **(K)** Inositol binding and uptake analysis of *Cryptococcus itr4∆, ITR4*^*OE*^*,* and *ITR4*^*Q388A, Q389A*^ strains. **(L)** Localization changes of *ITR4* in response of different carbon source. **(M)** Quantitative data of the percentage of cells with membrane localization. The data underlying this figure can be found in the supplemental information file S1 Data.

In addition, expression of the *ITR4*^*∆11-119*^ in the *C. neoformans itr4∆* mutant did not rescue the capsule defect phenotype, indicating a critical role for the N-terminal sequence in Itr4 function (Fig 4A). To test whether the intracellular fluorescent signal observed in the fluorescently tagged *ITR4*^*∆11-119*^ alleles due to protein misfolding and subsequential degradation, we monitored the stability of fusion proteins in a western blot. Our data showed that in the *S. cerevisae* strain expressing *ITR4*^*∆11-119*^*:GFP* allele, the fusion protein was stable (S5A Fig). However, we failed to detect the *ITR4*^*∆11-119*^*:mCherry* protein that was expressed in *C. neoformans itr4*∆ background (S5B Fig). This data indicate that the N-terminus-truncated Itr4 protein may not be stable, leading to mislocalized mCherry signal. We posit that the N-terminal sequence is essential for proper Itr4 dimerization, cellular localization and function. It remains to be studied whether this region may be required for sensing inositol and activating downstream signaling, a function similar to the C-terminal tail of Snf3 and Rgt2 in *S. cerevisiae* [31].

**Fig 4:**
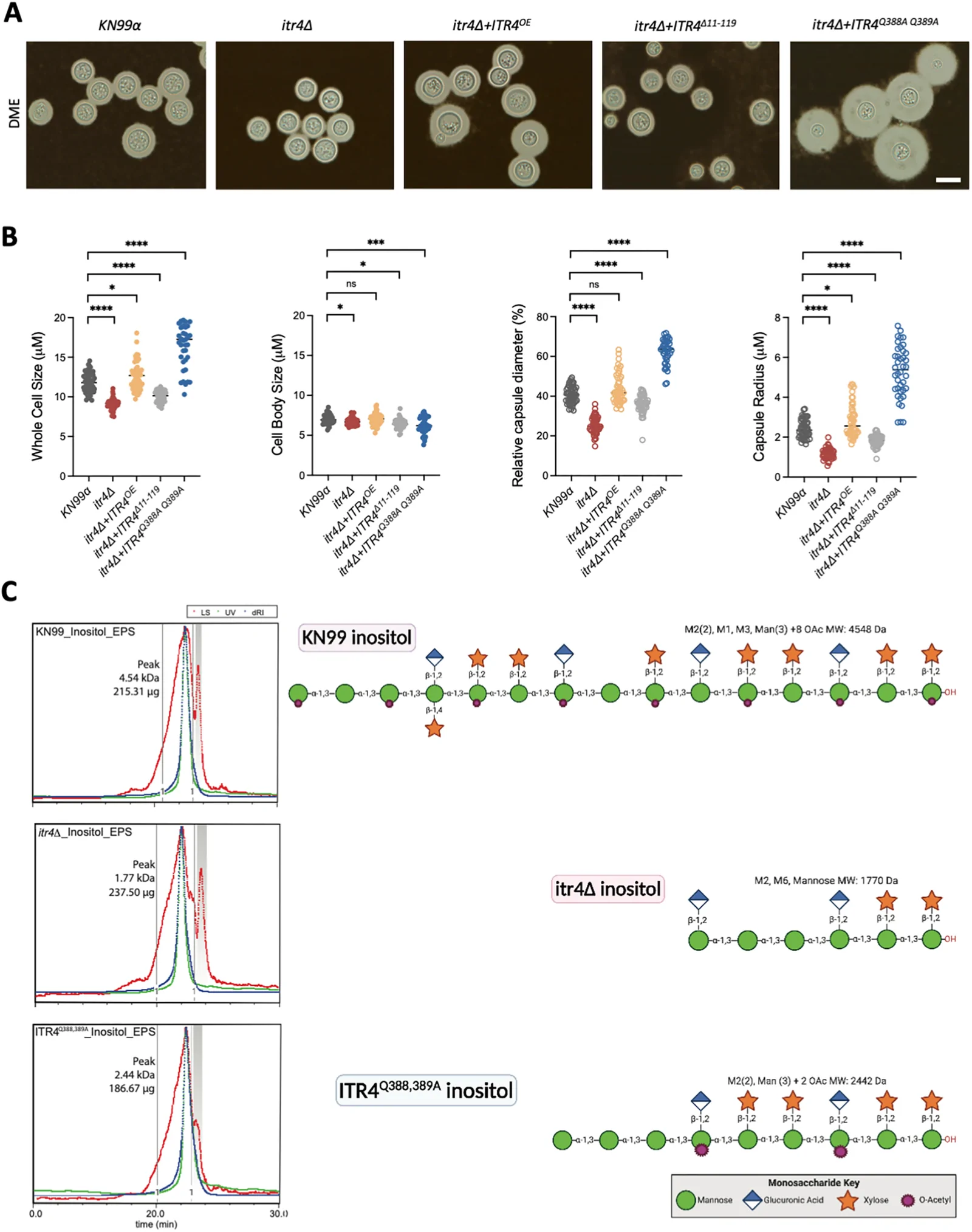
Itr4 regulates capsule size and structure. **(A)** Capsule formation of KN99**a**, *itr4∆, ITR4*^*OE*^*, ITR4*^*∆11-119*^ and *ITR4* ^*Q388A*^, strains was visualized by India ink staining after cells were grown on DME medium for 3 days. **(B)** Quantitative data of both cell size and capsule size from over 50 cells from each sample are presented. Statistical analysis was done using two-tailed Student *t* test. “****”, *P* < 0.0001. Bar, 10 µm. The data underlying this panel can be found in the supplemental information file S1 Data. **(C)** Characterization of GXM polymers in WT and the *itr4∆* mutant strains. SEC-MALS analysis of EPS from KN99**a**, *itr4∆,* and *ITR4* ^*Q388A, Q389A*^ cultured in inositol shows differences in size. GXM motif composition of EPS given reported KN99**a** motif expression. These structures are representative of but one of a number of ways these motifs could come together to make the polymer masses observed.

### Identification of key amino acids required for Itr4 function

Utilizing the Itr4 structural model, we further identified two predicted inositol binding sites (Q388 and Q389) (Figs 3C and S3A). The F497 residue was in close proximity to the ligand in the bulding pocket but not directly interact with inositol in our structural model, and was selected as a control. To determine their potential importance for Itr4 function, we generated a *ITR4*^*Q388A, Q389A*^ allele and a *ITR4*^*F497A*^ allele and expressed them in the *Sc itr1 itr2* heterologous system. Membrane localization was detected for both mutant alleles (Figs 3A and S4A). Inositol uptake assays showed that yeast strain expressing the *ITR4*^*Q388A, Q389A*^ mutation allele had increased levels of inositol uptake activity compared to the strain expressing the wild-type *ITR4* (Fig 3B), while the *ITR4*^*F497A*^ allele did not exhibit any difference (S4B Fig). These data indicate that these two glutamine amino acids may function as a gatekeeper to limit inositol transport, and their mutations partially open the channel.

To test this model in silico, we performed molecular dynamics (MD) simulation analysis to simulate the interaction between inositol and Itr4 and Itr4^Q388A, Q389A^ protein. Our data suggest that Q388 and Q389 are critical residues of inositol recognition and retention within the Itr4 transporter (Fig 3C–3H). Mutation on these residues decreases ligand stability in the binding pocket, facilitating inositol translocation through the transmembrane channel and supporting the role of the Q388A/Q389A variant as a transporter state that permits enhanced inositol passage across the membrane, which is consistent with the results of inositol uptake assay (Fig 3B). Our structure modeling also showed that the Q388A, Q389A mutation led to a large central cavity with lower interacting energy potential that may be more amenable to transport (S3 Fig). In addition, the Q388A/Q389A mutation also showed an increased frequency in forming hydrogen bond with inositol at Q262, suggesting a possibly more stable or persistent binding with inositol at that site (Fig 3E and 3H). It is likely the mutation also enhanced inositol binding at a secondary site, leading to stronger activation of downstream signaling. Therefore, the Q388A, Q389A point mutation produced a dominant active Itr4 allele that can both sense and transport inositol.

### The *ITR4*^*Q388A, Q389*A^ allele yields larger capsule but typical GXM particles

The functional importance of Itr4 N-terminus and the putative inositol-binding sites in *C. neoformans* were assessed using an mCherry-tagged strain for Itr4, under the control of its native promoter (*P*_*ITR4*_*-ITR4:mCherry*). Utilizing the Actin promoter, we also generated strains overexpressing *ITR4* (*P*_*ACT1*_*-ITR4:mCherry*), *ITR4*^*∆11-119*^ (*P*_*ACT1*_*-ITR4*^*∆11-119*^*:mCherry*) or *ITR4*^*Q388A, Q389A*^ (*P*_*ACT1*_*-ITR4*^*Q388A, Q389A*^*:mCherry*) alleles. The expression and stability of *ITR4*, *ITR4*^*∆11-119*^ and *ITR4*^*Q388A, Q389A*^ alleles were evaluated by the mCherry signal under fluorescence microscopy (Fig 3I) and direct protein detection in a western blot using anti-mCherry antibody (S5 Fig). While the strains expressing *ITR4* or *ITR4*^*Q388A, Q389A*^ retained membrane localization, the strain expressing the *ITR4*^*∆11-119*^ allele lost most of plasma membrane localization and instead most mCherry signal was localized in cytosol. Interestingly, detection of the Itr4^∆11-119^:mCherry protein using mCherry antibody was not successful, indicating that this protein may be unstable and could be misfolded and degraded (S5 Fig). The control strain expressing *P*_*ITR4*_*-ITR4:mCherry* manifested a weak plasma membrane fluorescence signal, suggesting a lower expression level compared to that under the control of the Actin promoter (Fig 3I and 3J).

We further measured inositol binding and uptake activities for the *itr4∆* strain expressing of the *ITR4* and *ITR4*^*Q388A, Q389A*^ alleles by using ^3^H-labeled *myo*-inositol at 0 and 30 °C as described previously [21]. Our results showed that the *itr4∆* strain had lower binding and uptake signals than wild type, while the *itr4∆* expressing *ITR4*^*Q388A, Q389A*^ alleles had significantly higher binding activity (Fig 3K). This result indicates that the *ITR4*^*Q388A, Q389A*^ allele may also enhance the inositol binding to the ITR4 ligand-binding site in addition to increasing inositol transport activity, thus promoting inositol signaling activation.

To investigate the Itr4 localization changes in response to different carbon sources, strains expressing *P*_*ITR4*_-*ITR4:mCherry* were grown in YPD overnight and reinoculated to YNB + 0.1% inositol, YNB + 1% inositol and YNB + 1% glucose media and incubated for 2 hours. Our data showed that, when *P*_*ITR4*_-*ITR4:mCherry*-expressing cells grown on YNB + 1% glucose, the mCherry fluorescence signal was weakly localized to the plasma membrane, and its expression increased when switched to YNB + 0.1% inositol medium. When cells were transferred to YNB + 1% inositol condition, the fluorescent signal also increased, but was detected mostly in cytosol, indicating an internalization of the Itr4:mCherry protein. The percentage of cells with membrane localization under different conditions was quantified (Fig 3L and 3M).

Consistent with our previous studies demonstrating that inositol can significantly affect the capsule [25], we found that the *itr4∆* cells produced smaller capsule sizes (Fig 4A). To understand how Itr4 regulates capsule growth, we examined capsule in the *itr4*Δ mutat and its *ITR4*^*OE*^, *ITR4*^*∆11-119*^ and *ITR4*^*Q388A, Q389A*^ expressing strains under in vitro capsule-inducing conditions [32]. We observed a significantly increased capsule size in the *ITR4*^*OE*^ and *ITR4*^*Q388A, Q389A*^ strains and a reduced capsule size in the *itr4*Δ and *ITR4*^*∆11-119*^ strain (Fig 4A and 4B). The large capsule size of the strain expressing the *ITR4*^*Q388A, Q389A*^ allele is consistent with our hypothesis that it is a dominant active allele.

We further analyzed capsule structural changes in the *itr4*∆ backgrounds by purifying the extracellular polysaccharides (EPS) secreted from KN99, *itr4*∆ and *ITR4*^*Q388A, Q389A*^ strains. Our 1D [^1^H] NMR measurement showed no change in EPS structure or Glucuronoxylomannan (GXM) O-acetylation pattern between KN99, *itr4*∆ and *ITR4*^*Q388A, Q389A*^ strains (S6 Fig). Further analysis by SEC-MALS (Size Exclusion Chromatography coupled with Multi-Angle Light Scattering) shows that while the structure of the EPS polymers did not change, the size of the polymers in both the *itr4∆* and *ITR4*^*Q388A, Q389A*^ strains were roughly half that of the parental strain (Fig 4C; S7 Table). In addition, the *ITR4*^*Q388A, Q389A*^ allele also showed less capsule density than either the parent or *itr4*∆ mutant. These data suggest that the EPS polymer size, rather than polymer structure, contributes to the changes in biological function of the *itr4*∆ mutant strains and their interactions with the host.

### Itr4 is required for *C. neoformans* to overcome the anti-fungal activity of macrophages

A key characteristic of *C. neoformans* as a facultative intracellular pathogen is its ability to survive and replicate within the phagolysosome of macrophages [33]. The capsule has antiphagocytic effects during the macrophage-pathogen interaction [34]. To investigate whether Itr4 was required for fungal cell survival in macrophages, we performed *Cryptococcus*-macrophage interaction assays using the macrophage-like cell line J774. After coincubation with *C. neoformans* for 4 h, J774 cells were fixed with methanol and stained with Giemsa staining, and the phagocytosis rate was determined by the number of yeast cells inside each J774 cell. Macrophage-like cells co-cultured with KN99**a** resulted in ~30% of J774 cells containing ingested yeasts, with ~2.0 yeast cells on average per host cell. These phagocytosis numbers increased when co-cultured with the *itr4∆* mutant or *ITR4*^*∆11-119*^, but decreased when co-cultured with *ITR4*^*OE*^ or *ITR4*^*Q388A, Q389A*^*-*expressing yeast cells, suggesting that Itr4 promotes phagocytic evasion (Fig 5A and 5B). We found that the expression of the Th1 cytokine IFN-γ was significantly increased in the *itr4*Δ, *ITR4*^*OE*^ and *ITR4*^*∆11-119*^ co-incubated macrophages (Fig 5C). These observations suggest that increased IFN-γ upon interaction is important during phagocytosis, which is consistent with our previously reported data [24].

**Fig 5:**
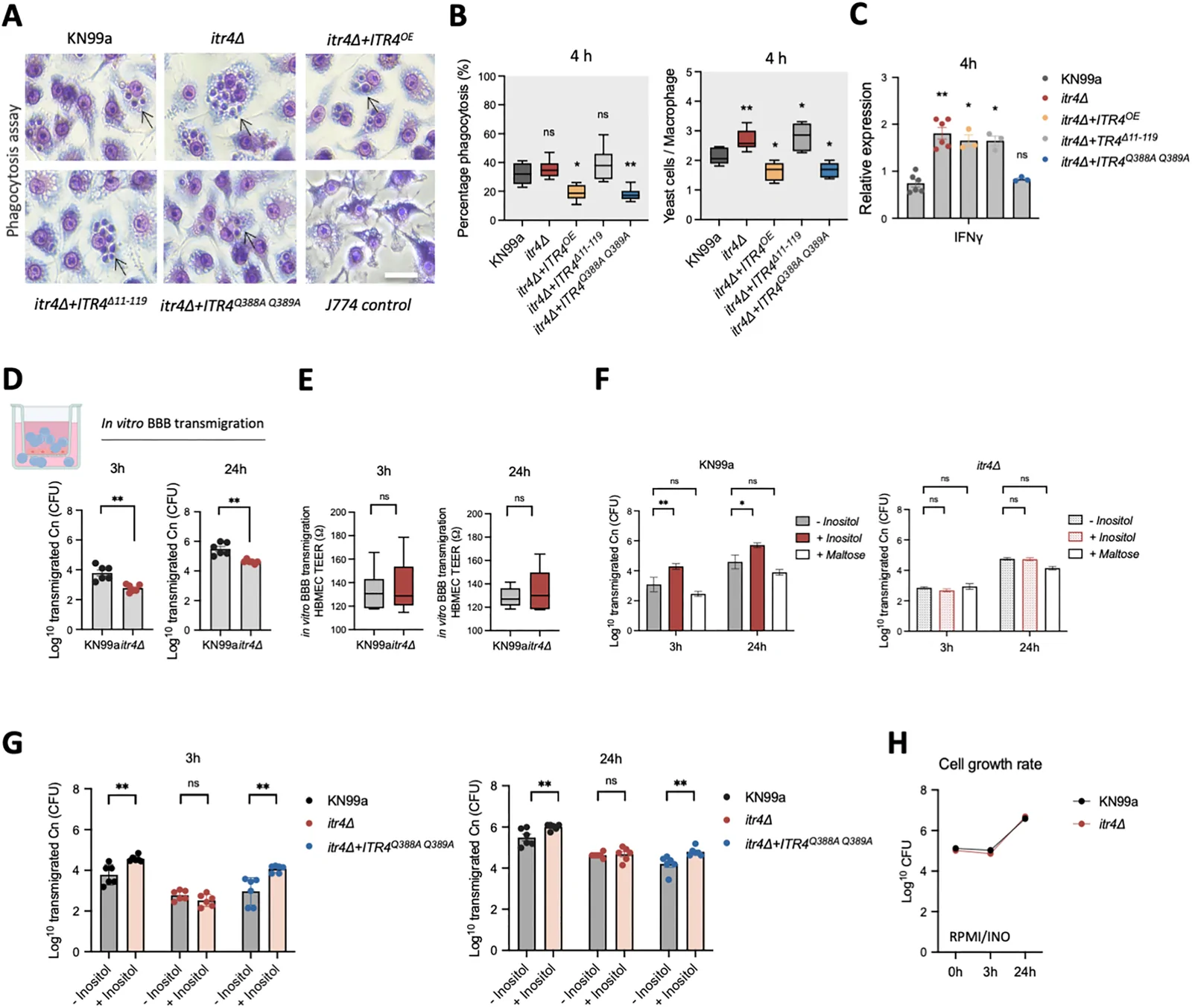
Itr4 is required for *C. neoformans* to overcome the antifungal activity of macrophages and cross the blood–brain barrier. **(A)** Typical field views of phagocytosis assay for each strain. **(B)** The phagocytosis rate of *C. neoformans* in the J774 macrophage cell line was measured in 48-well plates. PBS-washed wild-type KN99**a***, itr4∆, ITR4*^*OE*^*, ITR4*^*∆11-119*^ and *ITR4*^*Q388A, Q389A*^ strains were added to the activated macrophages and incubated for 4 h at 37 °C with 5% CO_2_. Cells were fixed with methanol and stained with Giemsa stain before counting. The percentage of macrophages containing internalized yeast cells was measured from over 100 macrophages. Error bars indicate standard deviations. “**”, *P <* 0.01. “*”, *P <* 0.05 (determined by Mann–Whitney test). **(C)** Cytokine gene transcription during phagocytosis of each strain. Differential gene expression relative to GAPDH was examined by qRT-PCR with cytokine-specific primers and calculated by the ΔΔ*CT* method. The data shown are cumulative from three independent experiments. “**”, *P <* 0.01 (determined by Mann-Whitney test). **(D)** In vitro BBB transmigration assay. Cryptococcal transmigration assay was done with an in vitro human BBB model generated with HBMEC as described in Materials and Methods. Transmigration rate of wild-type KN99**a** and *itr4*∆ mutant strains after 3 and 24 h incubation. Created in BioRender. Wang, Y. (2026) https://BioRender.com/ikr4wyf. **(E)** TEER resistance measurement of the Transwells with wild-type KN99**a** and *itr4*∆ mutant strains. **(F)** Transmigration assays were done with *C. neoformans* strains KN99**a** and *itr4*∆ mutant strains in the absence or presence of 1 mM inositol in the bottom compartment. An equal concentration (1 mM) of inositol or maltose was added to the bottom compartment prior to addition of fungal cells to the top compartment. Maltose was used as a different monosaccharide control. The number of *Cryptococcus* cells transmigrated was determined by plating the medium collected from the bottom compartment after 3 and 24 h incubation. **(G)** Transmigration rate of indicated strains in the absence or presence of 1 mM inositol after 3 and 24 h incubation. **(H)** The growth rate of wild-type KN99**a** and *itr4*∆ mutant strains in RPMI medium. The data underlying this figure can be found in the supplemental information file S1 Data.

### Itr4 is required for inositol-mediated fungal traversal across the blood–brain barrier in an in vitro model

Previously, we demonstrated that *C. neoformans* Itr1a and Itr3c are two major *ITR*s for inositol uptake and are required for dissemination across the BBB during brain infection [13]. To determine whether the inositol sensor Itr4 was required for fungal transversal across the BBB, we examined the transmigration ability of the *itr4∆* mutant in the in vitro BBB model as previously described [13]. In the absence of additional inositol, the *itr4∆* transmigration rate decreased by ~50% at 3 and 24 h incubation period compared to the wild type, indicating that Itr4 is required for cryptococcal crossing of the BBB (Fig 5D). Under this condition, the Transendothelial Electrical Resistance (TEER) measurement indicated that the membrane was intact (Fig 5E). With inositol treatment, cryptococcal traversal was enhanced regardless of the presence of *ITR4*; however, the *itr4∆* mutant showed significantly less transmigration than the parental control. The transmigrated wild-type cells increased by ~3- and ~5-fold in the presence of inositol at 3 and 24 h incubation, respectively, compared to in the absence of supplemental inositol. In contrast, the *itr4∆* transmigration rate was similar to the control (Fig 5F). We also tested the *ITR4*^*Q388A, Q389A*^ strain for its transmigration efficiency using the in vitro BBB model. Our data showed that this mutant had reduced transmigration, likely due to its enlarged capsule size, but an increased transmigration rate was observed in the presence of additional inositol (Fig 5G).

To address the concern that the CFU of different strains may be influenced by growth rate difference, we compared the growth rate of wild type and the *itr4∆* mutant in medium with or without addition of inositol. The results showed that all tested strains have similar growth rates regardless of the presence of additional inositol (Fig 5H). Thus, the data are consistent with our interpretation that Itr4 plays an important role in responding to inositol availability and contributes to cryptococcal traversal across the BBB.

### The *ITR4*^*Q388A, Q389A*^ allele shows attenuated virulence

In vitro analyses showed that Itr4 is important for capsule formation, mating, macrophage phagocytosis and BBB crossing, suggesting a potential role in host-pathogen interactions. We then examined the virulence of the *itr4*Δ, *ITR4*^*OE*^, *ITR4*^*∆11-119*^ and *ITR4*^*Q388A, Q389A*^ strains in a murine inhalation infection model. Our results showed that mice infected with *itr4*Δ, *ITR4*^*∆11-119*^, *ITR4*^*OE*^ or *ITR4*^*Q388A, Q389A*^ strain all manifested prolonged survival than mice infected with wild-type control, indicating a virulence attenuation (Fig 6A). These results indicate that the *ITR4*^*Q388A, Q389A*^ allele had significant virulence attenuation.

**Fig 6:**
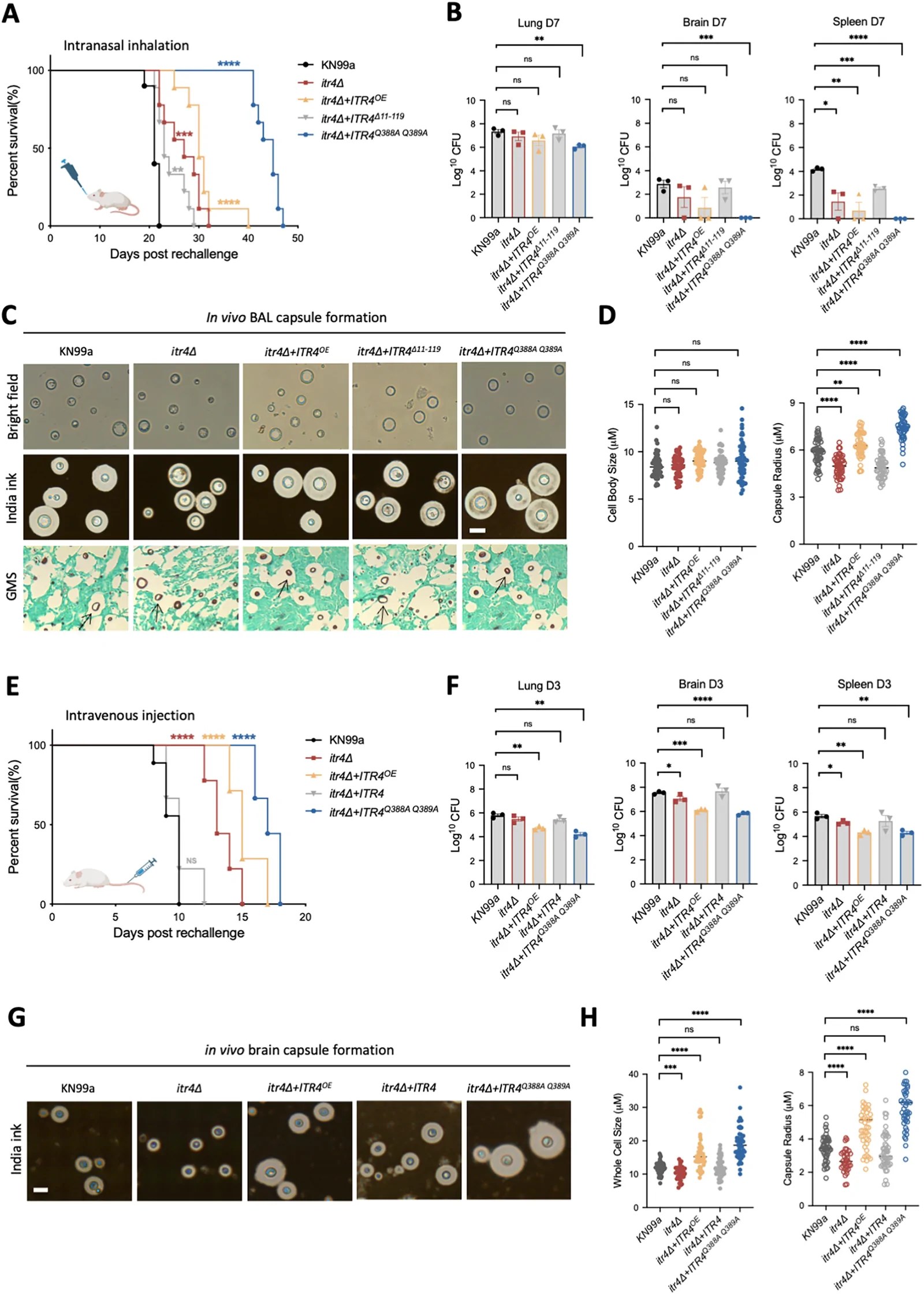
Itr4 is required for fungal virulence in animal models of systemic cryptococcosis. **(A)** Survival rates of wild-type KN99**a** and its *itr4∆, ITR4*^*OE*^*, ITR4*^*∆11-119*^ and *ITR4* ^*Q388A, Q389A*^ mutant strains in a murine intranasal inhalation model of systemic cryptococcosis. “****”, *P* < 0.0001; statistical analysis was done using a log-rank (Mantel-Cox) test. Created in BioRender. Wang, **Y.** (2026) https://BioRender.com/qpsdd8t. **(B)** Fungal burdens in the lungs, brains, spleens of animals infected with 1 × 10^4^ CFU of indicated strains at 7DPI. Each symbol represents one mouse. Bars represent the mean values ± standard errors of the means. “****”, *P* *<* 0.0001; “*”, *P* *<* 0.001 “**”, *P* *<* 0.01; “*”, *P* *<* 0.05 (determined by One-way ANOVA nonparametric test for multiple comparisons). **(C)** BAL fluid and histopathological examination during lung infection at 7 DPI. These fungal cells were examined under microscope with or without India ink staining, and the white halo surrounding yeast cells reflects the capsule. For histological examination, the lungs were fixed and stained with GMS. Fungal cells appear dark brown or black with GMS staining. Yeast cells are highlighted (black arrow). **(D)** Quantitative results of yeast cell body size and capsule size during lung infection. For each of the five strains, 50 cells were quantified and conducted for statistical analysis based on the microscopic images. Each error bar indicates the standard deviation among the five independent quantifications. “**”, *P* < 0.01; “***”, *P* < 0.001 (determined by *t* test). **(E)** Survival rates of indicated strains in a murine intravenous injec*t*ion infection model of systemic cryptococcosis. Balb/c mice were infected with 5 × 10^4^ yeast cells of each strain. “****”, *P* < 0.0001; statistical analysis was done using a log-rank (Mantel-Cox) test. **(F)** Fungal burdens in the lungs, brains, spleens of animals infected with 5 × 10^4^ CFU of indicated strains at 3 DPI. **(G)** Brain tissue examination during intravenous injection infection. Cryptococcal cells were recovered from infected brain tissues at 3 DPI. These fungal cells were examined under microscope with India ink negative staining. **(H)** Quantitative results of yeast cell size and capsule size in the infection site in the brain in vivo. Quantitative data from over 50 cells from each sample are presented. The data underlying this figure can be found in the supplemental information file S1 Data.

To better understand the mechanism of altered virulence of these *itr4* mutant strains, fungal burdens in lungs, brains, and spleens of 3 mice per group were examined at day 7 post-infection (7 DPI). While significantly reduced CFU was recovered in the lungs infected with the *ITR4*^*Q388A, Q389A*^ allele compared to wild-type infected ones, no CFUs were recovered in brains and spleens of those animals, indicating a lack of dissemination at this time point (Fig 6B). Histopathology results of infected organs demonstrated less severe lesion development in lungs and brains infected by the *itr4∆, ITR4*^*OE*^ or *ITR4*^*Q388A, Q389A*^ strain (S7 and S8 Figs), which are consistent with their virulence attenuation. At the endpoint, while all mice contained high levels of CFUs in the infected lungs, several of the *itr4∆, ITR4*^*OE*^, *ITR4*^*∆11-119*^ and *ITR4*^*Q388A, Q389A*^ strains were cleared of their brain or spleen infection, with some of them containing low levels of fungal CFUs compared to wild-type control group (S8 Fig).

We isolated yeast cells from bronchoalveolar lavage fluid (BALF) of mice infected with different strains and analyzed their capsule size to examine the role of Itr4 in capsule size regulation during infection. Compared to wild-type KN99**a**, we detected significantly increased capsule size in *ITR4*^*OE*^ and *ITR4*^*Q388A, Q389A*^, but smaller capsule in *itr4*∆ and *ITR4*^*∆11-119*^ cells isolated from infected mice, which is consistent with the observations at in vitro conditions (Fig 6C and 6D). Altogether, these data confirm that Itr4 is involved in capsule size regulation during infection in vivo, and a significantly enlarged capsule may have slowed down the yeast dissemination, leading to virulence attenuation.

To understand how these capsuel changes in different strain background impact host immune responses, we analyzed the expression of chemokine genes in lung tissues infected with different strains at 7 DPI by qRT-PCR. Uninfected C57BL/6 mice were analyzed as a control. We found that the expression of IFN-γ was significantly increased in the lungs of mice infected with *itr4*Δ, and significantly decreased in *ITR4*^*Q388A, Q389A*^ infected mice (S7A Fig). These observations suggest that increased influx of IFN-γ upon infection with the *itr4*Δ mutant is associated with containment of the infection, which is consistent with our previously reported data [24]. Our lung gene expression analysis also showed that genotype-dependent differences in cytokine and macrophage polarization gene expression coincided with fungal burden and survival outcomes. Compared to the parental strain, *itr4*Δ infected lungs showed higher expression of IFN-γ, IL-12a, IL-12b, and TNF-α, followed by increased *CCR2* and *NOS2* expression but decreased *ARG1* expression. In contrast, the *ITR4*^*Q388A, Q389A*^ strain showed reduced expression of *CCR2* and *NOS2* genes, with lower *ARG1* and IL-10 levels. IL-4 expression remained high across groups (S7A Fig). Together, these data indicate that differential modulation of lung innate and chemokine responses distinguishes Itr4 alleles and aligns with their respective CFU and survival phenotypes in animal models.

### Involvement of *Itr4* in in vivo brain infection in a mouse intravenous model

Given that Itr4 was required for BBB crossing in our in vitro BBB model (Fig 5D and 5F), we evaluated the involvement of Itr4 in BBB crossing in vivo by infecting mice intravenously with the *itr4*Δ, *ITR4*^*OE*^, and *ITR4*^*Q388A, Q389A*^ strains, respectively. All these manifested significant virulence attenuation (Fig 6E), consistent with our observation in the inhalation animal infection model.

To determine the rate of fungal transversal across the BBB to cause brain infection in the intravenous infection model, lungs, brains, and spleens from mice injected with different *itr4* mutants were isolated at 3 DPI and their fungal burdens determined by yeast CFUs. Our results showed that there was a significant difference in fungal burden in the brain between mice infected by wild-type and the *itr4*Δ mutant at 3 DPI. On the other hand, the fungal burden in all three organs was significantly decreased in *ITR4*^*OE*^ and *ITR4*^*Q388A, Q389A*^ infected mice (Fig 6F). Our results thus demonstrate that the inositol transceptor Itr4 is required for fungal cells to either cross the BBB or grow in the brain after transmigration.

To examine the role of Itr4 in capsule size regulation during CNS infection, we homogenized the brain tissues infected with different strains and analyzed their capsule size. Similar to what we have observed in vitro conditions, we detected significantly enlarged capsule of the *ITR4*^*OE*^ and *ITR4*^*Q388A, Q389A*^ cells isolated from infected mouse brains, while the capsule size was smaller in the *itr4*∆ mutant (Fig 6G and 6H). Histopathology of infected mouse brain sections also indicated that the *ITR4*^*OE*^ and *ITR4*^*Q388A, Q389A*^ capsules were larger than the wild-type and *itr4*∆ mutant strains (S7 and S8 Figs). Altogether, these results further confirmed that fungal Itr4 plays an important role in capsule size regulation and fungal pathogenesis, including *Cryptococcus* traversal of the BBB in CNS infection.

### Itr4 regulates genes involved in inositol metabolism during inositol incubation

To further understand how Itr4 regulates *Cryptococcus* cells responding to inositol, we performed RNA-Seq assays to analyze transcriptional profiles of the *Cryptococcus* wild-type KN99**a** and *itr4*∆ mutant that were either treated with or without inositol for 24 h. Our RNA-seq data identified a large number of differentially expressed genes (DEG) when compared between KN99**a** and *itr4*∆ under YNB with either only 1% glucose or both 1% glucose and 1% inositol presence (Fig 7A). Analyzing the differentially expressed genes (DEGs) using Ingenuity Pathway Analysis (IPA) identified several pathways that were upregulated by Itr4. A KEGG (Kyoto Encyclopedia of Genes and Genomes) pathway enrichment analysis highlighted the overrepresented pathways, including: ‘Carbon metabolism’ (map01200, *p* = 0.0018), ‘Valine, leucine and isoleucine degradation’ (map00280, *p* = 0.0022), ‘Citrate cycle (TCA cycle)’ (map00020, *p* < 0.0087), ‘Pyruvate metabolism’ (map00620, *p* = 0.0117), and ‘Cell cycle - yeast’ (map04111, *p* = 0.0155) (Fig 7C). A GO enrichment analysis plot (Fig 7D) revealed the top Biological Process (BP) terms include ‘DNA replication’ (GO:0006260, *p* < 0.01),‘intracellular signal transduction’ (GO:0035556, *p* < 0.05), and ‘organic acid metabolic process’ (GO:0006082, *p* < 0.01).

**Fig 7:**
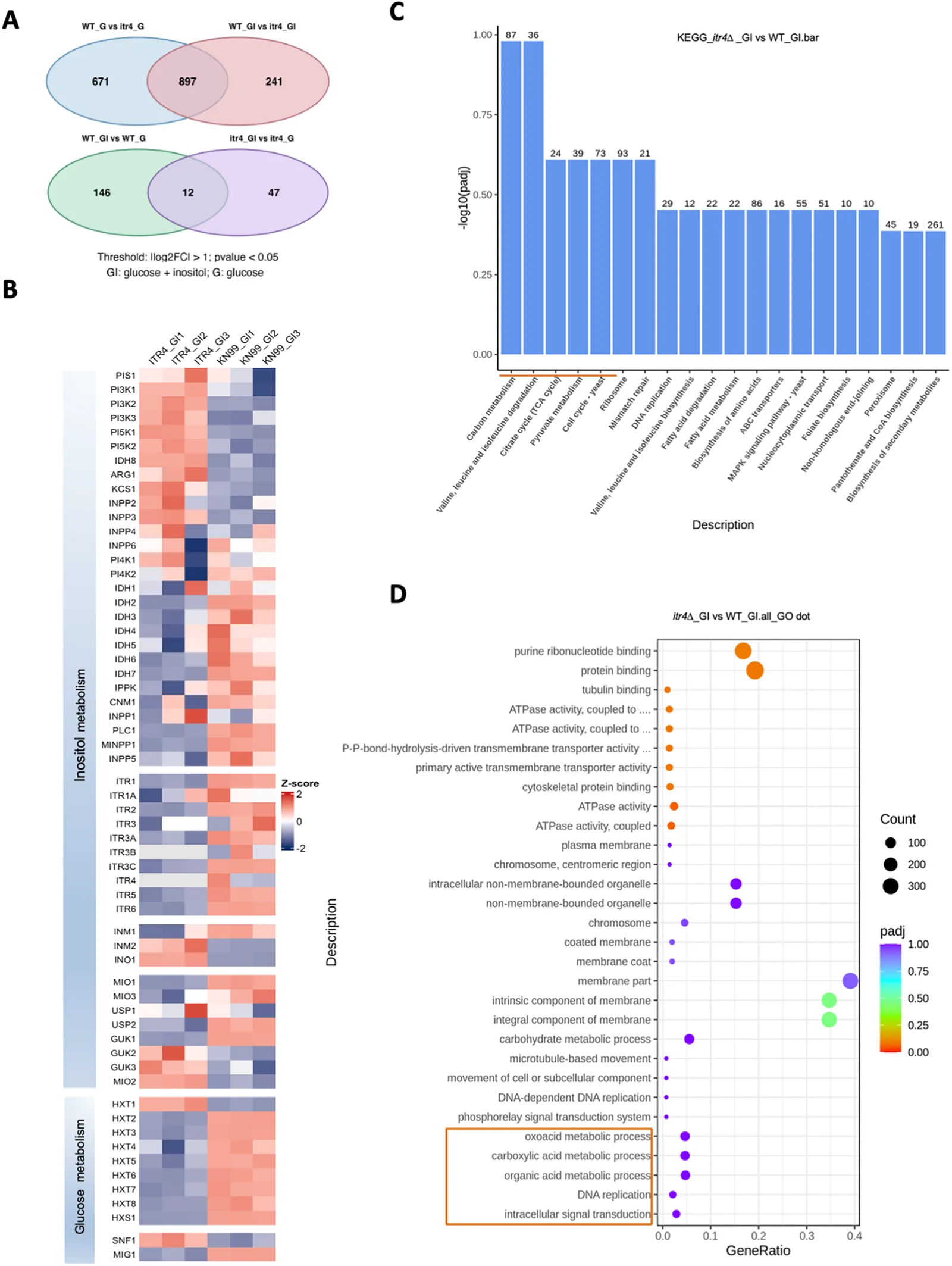
Functional enrichment analysis of differentially expressed genes in the *itr4Δ* mutant. **(A)** Venn Diagram of Differentially Expressed Genes (DEGs) (|log2 FC| > 1, *p* < 0.05) identified from RNA-seq analysis between the *itr4∆* mutant and wild-type KN99**a** under two different carbon conditions: YNB + 1% glucose + 1% inositol (GI) and YNB + 1% glucose (G). Venn diagram illustrates DEG distribution and overlap. Overlapping DEGs represent *ITR4*-dependent changes consistent across glucose-containing conditions, while unique DEGs in glucose/inositol reflect inositol-specific responses. **(B)** Heatmap showing relative gene expression in *itr4*∆ mutant and KN99**a** samples under glucose/inositol (GI) conditions across three biological replicates. Expression values were normalized to gene-wise Z-scores across the six samples, with blue and red indicating relatively lower and higher expression, respectively. Genes are grouped by pathway and separated by blank spacing. Gray hatched cells indicate genes for which expression was not detected. The data underlying this panel can be found in the S8 Table. **(C)** Bar plot of KEGG pathway enrichment analysis from RNA-seq data. Y-Axis (−log10(p.adj)) measuring statistical significance of pathway enrichment, adjusted for multiple testing (p.adj). Higher values indicate greater significance. X-Axis (Pathway Descriptions) listing enriched KEGG pathways, ordered from left (most significant) to right (least). Bar length representing −log10(p.adj), longer bars denote higher significance. Numbers on bars indicating the gene count and how many differentially expressed genes map to each pathway. Larger counts suggest broader involvement. **(D)** GO enrichment dot plot of top terms across Biological Process (BP), Molecular Function (MF), and Cellular Component (CC) categories. GO dot plot of selected terms, with dot size indicating gene count and color gradient showing significance. X-axis (Gene Ratio) represents the proportion of differentially expressed genes in each term relative to the total genes annotated to that term. Higher ratios indicate a larger fraction of the pathway/term is impacted by the mutation. Dot Size (Count): Scales with the number of genes in the term. Larger dots mean more genes contribute to the enrichment, suggesting widespread effects. Dot Color (p.adjust) indicates adjusted *p*-value for statistical significance. Data from three biological replicates; enrichment performed using clusterProfiler (GO) and Pathview (KEGG) with H99 annotations from FungiDB.

When analyzing the genes involved in inositol function, we found that genes involved in inositol pyrophosphate biosynthesis and inositol de novo biosynthesis were mostly upregulated in the *itr4*Δ mutant background, while many genes involved in inositol uptake and inositol catabolism were down-regulated (Fig 7B; S8 Table). The Itr4 regulation of other *ITR* genes observed in RNA-seq data is consistent with our qRT-PCR data (Fig 2H). *Cryptococcus* can use inositol as a carbon source. Conversion of inositol to glucuronic acid by inositol oxygenases (*MIOs*) is the first step of the only known pathway for inositol catabolism [25]. We found that the gene encoding inositol oxygenase 1 (*MIO1*) was induced significantly less by inositol treatment in *itr4*∆ than in KN99**a** (Fig 7B; S8 Table). These gene expression data support a role for Itr4 in regulating the expression of genes involed in inositol acquisition and inositol catabolism.

Interestingly, the glucose transceptor homolog gene *HXS1* (High affinity glucose transporter, CNAG_03772) [35] was significantly repressed (>125-fold) in the *itr4*∆ cells, so was the *MIG1* homolog, while the *SNF1* homolog was significantly induced. Because *MIG1* and *SNF1* are two key carbon catabolite repression (CCR)-associated genes in *S. cerevisiae*, our data indicate that Itr4 may also regulate glucose signaling. Indeed, several hexose transporter homologs were also differentially expressed in wild-type and *itr4*∆ cells (Fig 7B; S8 Table). In addition, *SCW1* (cell wall integrity protein 1, CNAG_05663) [36], a gene required for cell wall integrity, and *BAT1* (Branched-chain-amino-acid transaminase, CNAG_05664) [36], a gene involved in amino acid metabolism, were completely silenced in the *itr4*∆ mutant background under the same culture condition (S4 Table). These data may indicate a connection between inositol sensing and glucose uptake, cell wall integrity and amino acid metabolism, suggesting a crosstalk among different metabolic pathways, and underscores the dynamic role inositol plays in the biology of *Cryptococcus*.

## Discussion

Pathogenic microbes must sequester nutrients from their hosts to support their proliferation and expression of virulence traits. We previously showed that *C. neoformans* can exploit the abundant inositol present in the brain for its development and virulence, supporting the progression of cryptococcal meningoencephalitis [12,13,21]. *C. neoformans* is an environmental fungus that occupies diverse ecological niches, such as plants and soils, where it interacts with amoebae. Since humans and animals serve as terminal hosts for *C. neoformans*, it has been proposed that the evolution of its virulence traits, and its capacity to infect human and animal hosts likely stems from adaptations acquired in its natural environments that also endow it with the capacity to resist clearance by the immune system. Previously, we demonstrated that *Cryptococcus* has a highly sophisticated inositol utilization system, enabling it to efficiently acquire and metabolize inositol, which is abundant in plants such as *Eucalyptus* species, to complete its sexual cycle. This provides a potential explanation how this fungus produces infectious spores in nature [9,11]. Interactions between *Cryptococcus* and soil amoebae (*Acanthamoeba castellanii*) have been shown to drive the development of virulence factors and confer the ability to survive within macrophages in mammalian hosts [37–39]. Similarly, the bacterial pathogen *Legionella pneumophila* can sequester inositol from amoebae, and its inositol metabolism promotes infection of both amoebae and macrophages [40]. It is conceivable that *C. neoformans* also senses and utilizes inositol in amoebae to support the development of fungal virulence factors, which helps survive predation. However, the mechanism by which *Cryptococcus* senses and responds to host inositol availability in the environment and in animal hosts was previously unknown.

In this study, we identified an inositol sensor, Itr4, which likely evolved from a transporter ancestor. Our data suggests that Itr4 enables the fungus to detect environmental inositol, thereby regulating the expression of genes involved in inositol acquisition and metabolism. This adaptive strategy, that presumably originally evolved for survival in the environment is also employed to efficiently sense and metabolize the abundant inositol found in the human brain. Therefore, we propose that this evolutionary specialization that once promoted survival in environmental hosts now facilitates infection of human hosts and that Itr4 serves as a critical sensor in this process (Fig 8). To our knowledge, this is the first defined inositol sensor identified in eukaryotes. Whether other fungi possess a similar inositol sensor remains to be determined.

**Fig 8:**
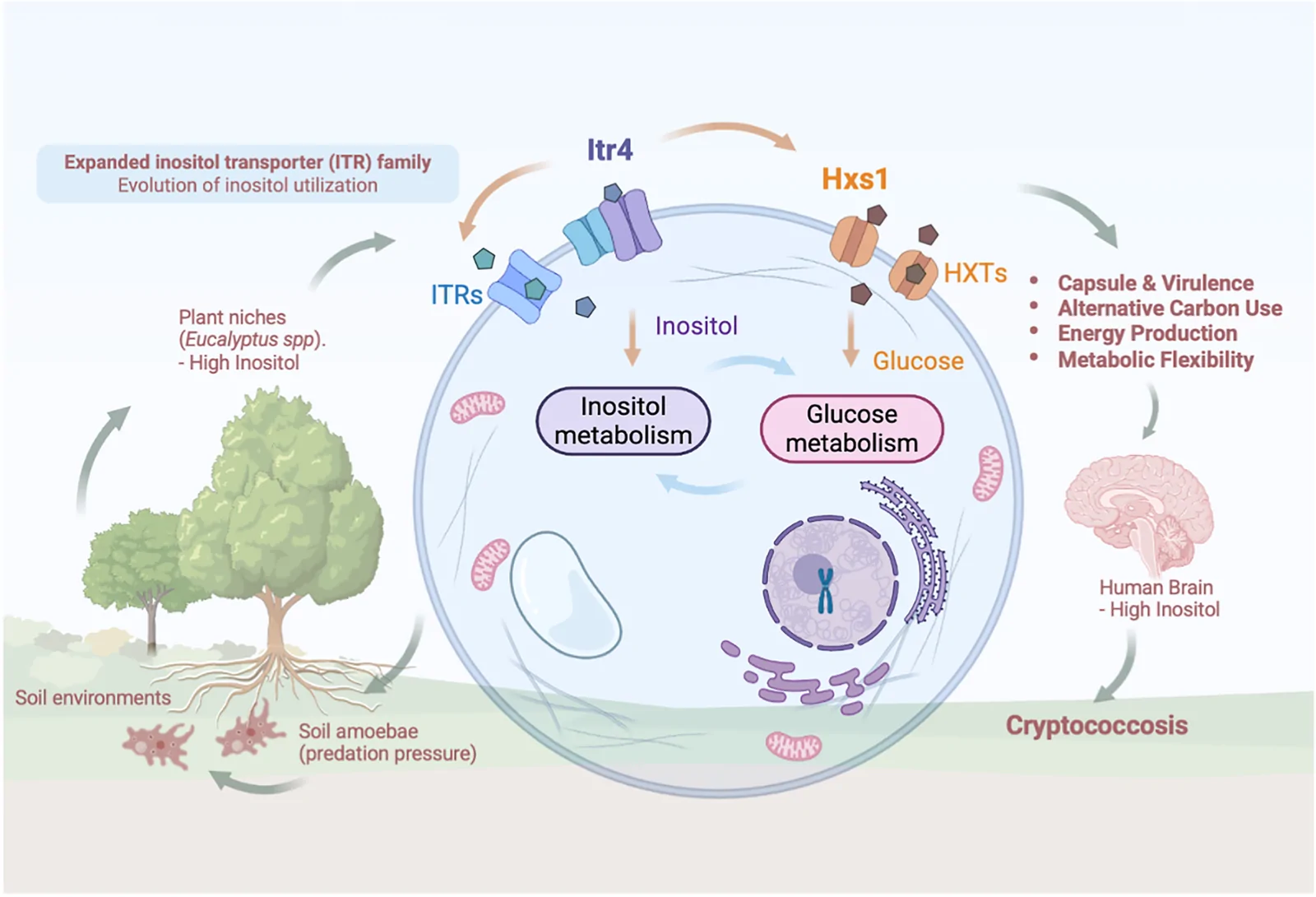
Proposed model for the role of Itr4 in coordinating carbon utilization and fungal virulence. Itr4 as an inositol receptor that is required to coordinate inositol and glucose metabolism by regulating ITRs and glucose sensor Hxs1. Itr4 regulates fungal pathogenesis in part through its regulation of capsule development. Itr4 serves as a critical sensor in the process of evolutionary specialization in this model. Created in BioRender. Wang, Y. (2026) https://BioRender.com/z8h6fdo.

Although glucose is the preferred sugar and energy source for many pathogens, *C. neoformans* can efficiently take up and utilize free inositol, which is abundant in the brain, for its development and virulence, through its expanded 10 member inositol transporter (*ITR*s) family and three member inositol oxygenases (*MIOs*) [13,21,25]. Transcriptomics studies have also shown that *ITRs* are upregulated in the cerebral spinal fluid (CSF) of patients with disseminated cryptococcal infections [41]. The identification of inositol sensor Itr4 in *C. neoformans* provides a mechanism on how the fungus senses and coordinates the function of this large family of *ITR*s. In this study, we further delineated the multifaceted role of Itr4 function, revealing its significance as a plasma membrane inositol transporter-like sensor (transceptor) and key regulator of inositol acquisition and metabolism. Our qRT-PCR and RNA-Seq data demonstrated that Itr4 regulates the expression of other *ITR*s, as well as genes involved in inositol metabolism (e.g., *MIO1*) in *C. neoformans*.

Sugar transceptors have been characterized in other fungi, including the well-studied glucose sensors Snf3 and Rgt2 in *S. cerevisiae* [31,42]. These two sensors work in concert in the yeast carbon catabolite repression (CCR), when extracellular glucose levels are high Rgt2 is expressed and Snf3 is repressed, leading to expression of low-affinity hexose transporter (e.g., Hxt1). Whereas in low glucose conditions Rgt2 is not required and Snf3 is expressed inducing high-affinity hexose transporters (e.g., Hxt2) expression [43]. Interestingly, while both Snf3 and Rgt2 have long C-terminal tails required for signal transduction, Itr4 contains a long N-terminal region essential for both membrane localization and function (Fig 3), with the *ITR4*^*∆11-119*^ allele failing to localize to the membrane and exhibiting the same capsule defect as that of *itr4*∆ (Fig 4). The AlphaFold structural modeling indicates that this region is required for Itr4 forming homodimer (S2 Fig). We found that the Itr4 lacking this N-terminus was either lowly expressed or unstable as we did not detect a specific protein band in a western blot (S5 Fig). Therefore, the specific function of this N-terminal region remains to be investigated. Based on structure modeling, we identified two amino acids required for Itr4 function. The *ITR4*^*Q388A, Q389A*^ mutant allele showed increased inositol uptake activity, suggesting that those two glutamines prevent inositol transport into the cell by locking it in the channel. The Q-A Itr4 mutations result in functional inositol transport, leading to a dominant dual functional inositol sensor. The ammonium permease Mep2 is another transceptor with known dual function to sense and transport ammonium in yeast [44,45]. Reported Mep2 protein structure revealed that the C-terminal region (CTR) is required for blocking ammonium transport [46]. Together, these data indicate that Itr4 can sense but not transport inositol due to glutamine-mediated channel restriction. It would be interesting to evaluate the function of similar amino acids in Snf3 and Rgt2 to see whether certain mutations could revive their glucose transporter function.

Through its receptor functions, we found that Itr4 is also involved in modulating the host response to *Cryptococcus* [25]. Our previous studies have demonstrated that inositol sensing and utilization influence capsule size and structure [24] which can, in turn, alter host responses like phagocytosis and pro-inflammatory IFNγ cytokine signaling. IFNγ plays an important role in host defense against *Cryptococcus* infection by regulating inflammation, activating macrophages and inducing T cell activation. In a recent GWAS of clinical isolates collected in Uganda [23,24], we identified differences in inositol-related phenotypes*. ITR4* was one of several genes identified as having polymorphisms associated with differential human immune responses and patient survival. These results from clinical isolates further confirm the importance of the inositol sensor Itr4 in regulating fungal virulence and disease outcome in patients. Subsequent mouse model GWAS studies identified alleles in *ITR4* and a few other genes associated with increased production of IFNγ. We found that *itr4∆* enhanced IFNγ production, while *ITR4* overexpression reduced IFNγ production (S7A Fig).

Consistent with Itr4 playing a regulatory role in fungal virulence, the *ITR4*^*Q388A, Q389A*^ mutation results in dramatically enlarged capsules while the *itr4∆* capsule is significantly smaller than wild type. The *ITR4*^*Q388A, Q389A*^ mutant also shows significantly decreased phagocytosis and elicited reduced IFNγ production while *itr4∆* elicited increased IFNγ production. Further, in our in vitro transmigration assay and in vivo animal experiment, *itr4*Δ shows reduced BBB penetration and attenuated CNS dissemination, leading to delayed meningitis and prolonged survival. Surprisingly, both the *itr4∆* and the *ITR4*^*Q388A, Q389A*^ mutation result in attenuated virulence via intranasal and intravenous modes of infection. These findings support a model in which a balanced Itr4 activity is required for its regulation of optimal capsule size and functionality for infection and pathogenicity [25,47]. Similar observations was reported in the study of Kar1 function in *S. cerevisiae* [48], and also in yeast overexpression strain collection analysis [49,50]. Our previous studies have also observed such paradoxical effect of some proteins in *C. neoformans* [51,52]. These data led us to conclude that proper inositol sensing through Itr4 influences the capsule surface and subsequently both immune response and disease outcome.

Our RNA-Seq data detected both glucose metabolism and inositol metabolism activities under a medium condition rich in both sugars, suggesting regulation of sugar metabolism in *C. neoformans* is distinct from the classic carbon catabolte repression (CCR) model. It is established that yeasts like *S. cerevisiae* will utilize glucose before utilizing other carbon availability in the same medium through a glucose repression pathway that involves the glucose sensors Snf3 and Rgt2, kinase Snf1, and transcription repressor Mig1 [53,54]. However, our data suggests *C. neoformans* deviates from this model as fungal inositol metabolism remains active even in the presence of abundant glucose. The shift from glucose to inositol metabolism in *Cryptococcus* likely requires a nutrient sensor to determine the prevalence of inositol and activate transcription of the transporters. The cryptococcal hexose transcepter-like protein Hxs1 functions as a high-affinity glucose transporter with possible sensory function [35]. Our data shows that *ITR4* expression is induced while *HXS1* expression is downregulated in the presence of inositol even when abundant glucose is also present. This metabolic flexibility, distinct from *S. cerevisiae*’s strict glucose repression, likely enhances *C. neoformans*’s host adaptation.

In aggregate, Itr4 is a multifunctional transceptor critical for inositol sensing, cellular regulation, and pathogenicity in *C. neoformans*. Its loss triggers a cascade of molecular and phenotypic changes, revealing novel links between nutrient sensing, host adaptation, and fungal virulence (Fig 8). Itr4 integrates glucose-inositol sensing to regulate capsule, phagocytosis, and virulence. These results expand our knowledge on the function of *C. neoformans ITR* family and highlight the unique contribution of Itr4 in nutrient sensing, host adaptation and fungal pathogenesis.

## Materials and methods

### Ethics statement

All studies were conducted following biosafety level 2 (BSL-2) protocols and procedures approved by the Institutional Animal Care and Use Committee (IACUC) of Rutgers University under protocol 999901066. Animal studies were compliant with all applicable provisions established by the Animal Welfare Act and the Public Health Services (PHS) Policy on the Humane Care and Use of Laboratory Animals.

### Strains, media, and growth conditions

*C. neoformans* strains used in this study are listed in S1 Table. *S. cerevisiae* strains and plasmids used in this study are listed in S2 Table. All primers used in this study are listed in S3 Table.

Strains were grown at 30 °C on yeast extract-peptone-dextrose (YPD) agar medium and synthetic (SD) medium. YNB medium without inositol was purchased from MP Biomedicals. Niger-seed medium was used to test for melanin production. Dulbecco modified Eagles (DME) medium for assessing capsule production was prepared as previously described [9,55]. A V8 juice agar medium (50 mL V8 original juice, 0.05% KH_2_PO_4_, and 2% agar, pH 5.0) and a modified Murashige and Skoog (MS) medium minus sucrose (Sigma-Aldrich) were used for mating and sporulation assays. Minimal medium (MM, 15 mM D-glucose, 10 mM MgSO_4_, 29.4 mM KH_2_PO_4_, 13 mM Glycine, and 3.0 μM Thiamine, pH 5.5) was used for capsule induction. All other media were prepared as described previously [26,56,57].

### Generation of *itr4∆*, *itr5∆* and *itr6∆* mutants

Mutants for *ITR4, ITR5* and *ITR6* were generated in both KN99α and KN99**a** strains backgrounds by overlap PCR as previously described [58]. The 5′ and 3′ regions of the *ITR4* gene were amplified from KN99α genomic DNA with primer pairs CX1005 and CX1006, and CX1007 and CX1008, respectively. The 5′ and 3′ regions of the *ITR5* gene were amplified from KN99α genomic DNA with primer pairs CX998 and CX999, and CX1000 and CX1001, respectively. The 5′ and 3′ regions of the *ITR6* gene were amplified from H99 genomic DNA with primer pairs CX991 and CX992, and CX993 and CX994, respectively (S3 Table). Each target gene replacement cassette was generated by overlap PCR. Purified overlap PCR products were precipitated onto 10 µl gold microcarrier beads (0.6 µm; Bio-Rad), and strains KN99α or KN99**a** were ballistically transformed as described previously. Stable transformants were selected on YPD medium containing G418 (200 mg/L) or NAT (100 mg/L). To screen for *itr4∆*, *itr5∆ or itr6∆* mutants, diagnostic PCR was performed by analyzing the 5′ junction of the disrupted mutant alleles with primers CX1009 and JH8994, CX1002 and JH8994, CX995 and JH8994. Positive transformants were identified by PCR screening with primers CX1010 and CX1011, CX1003 and CX1004, CX996 and CX997, respectively.

To generate complemented strains of the *itr4*Δ single mutant, a genomic DNA fragments that contained a 1.5 kb upstream region of *ITR4* ORF, the *ITR4* ORF, and its 500 bp downstream region was amplified by PCR with primers CX1222/CX1223. The *ITR4* PCR fragment was inserted into vector pJAF13 using in-fusion cloning. The construct *pJAF13-ITR4* was ballistically transformed in *itr4∆* mutant strains. The selecting colonies grown on YPD with both nourseothricin and G418 were further confirmed by PCR. Phenotypic assays were performed to identify transformants in which the *itr4*Δ phenotype was complemented.

To generate overexpression strain of the *itr4∆* mutant, the *ITR4* full-length genomic DNA was amplified with primers CX1800/CX1801 (S3 Table). The fragment was cloned into the BamHI site of vector pCXU200 containing the *Cryptococcus* actin promoter to generate a plasmid which contains *ITR4:mCherry* fusion fragment*.* Linearized plasmid was introduced into *itr4*Δ mutant strain to generate overexpression strain that expresses *Itr4:mCherry* protein.

### Generation of triple mutants and complemented strains

In mating assays, *C. neoformans* cells of opposite mating types were mixed and co-cultured on MS agar medium at 25 °C in the dark for 10 days and filamentation was examined by light microscopy. Spore production was visualized by microscopy and photographed. Basidiospores were dissected from mating assays performed on MS medium.

To generate *itr4∆ itr6∆* double mutants, a mating assay between α *itr4∆::NAT* and **a** *itr6∆::NEO* mutants was conducted and spores were isolated with an MSM system (Singer Instrument, England). Genomic DNA was isolated from all progenies that grew on YPD with both nourseothricin and G418. PCR was used to screen for *ITR4* gene deletion with primers CX1009/JH8994 and CX1010/CX1011, and for *ITR6* gene deletion with primers CX995/JH8994 and CX996/CX997 (S3 Table). To generate *itr4∆ itr5∆ itr6∆* triple mutants, a mating assay between α *itr4∆::NAT itr6∆::NEO* double mutant and **a** *itr5∆::NEO* mutant was conducted and spores were isolated. Triple mutants were screened first by PCR on cultures initiated from single spores. All mutants were confirmed by PCR. The mating type of each confirmed mutant strain was determined by PCR using mating type-specific primers and by genetic crossing.

### Assays for melanin and capsule production

Assays for melanin and capsule production were performed as previously described [55,59]. In brief, melanin production was assayed by inoculating *C. neoformans* strains into 2 ml of YPD liquid medium, incubating the culture overnight at 30 °C and spotting 5 µl of each culture with 10 times series dilutions on Niger-seed agar medium. The agar plates were incubated at 30 or 37 °C for 2 days, and pigmentation of fungal colonies was assessed and photographed. To examine capsule production, 30 µl of overnight cultures were inoculated on DME agar medium and incubated at 37 °C for 3 days. Capsule was visualized with India ink negative staining and observed with a 100× Olympus CX41 equipped with an Infinity digital camera (Olympus). Capsule sizes and cell body sizes of more than 100 cells were measured and calculated.

### Assays for stress responses and cell integrity

Each strain was incubated overnight at 30 °C in YPD and diluted to an optical density at 600 nm (OD600) of 2.0. The cells were washed, resuspended, and serially diluted (1:10) in dH_2_O and spotted (5 μl) on YPD agar plates containing 1.5 M NaCl, or 1.0 M KCl for osmotic shock, or 2.5 and 5.0 mM H_2_O_2_ for oxidative stress. To test cell integrity, cells were also spotted on YPD agar plates containing 0.05% SDS, 0.03% SDS, 0.5% Congo Red, or 250 μg/ml CFW. Plates were incubated at both 30 and 37 °C for two days and photographed.

### Inositol uptake assay

The inositol uptake assay was performed following a previously published method [19,20]. In brief, the *Cryptococcus* strains were grown in YPD liquid cultures overnight at 30 °C. Cells were diluted in YPD to an OD_600_ of 1.0, grown at 30 °C, and collected at an OD_600_ of 5.0 by centrifugation at 2,600*g* for 5 min. Cells were then washed twice with PBS at 4 °C and resuspended in 2% glucose to reach a final concentration of 2 × 10^8^ cells/ml as determined by the use of a hemacytometer. For the uptake assay, the reaction mixture (200 l) contained 2% glucose, 40 mM citric acid-KH_2_PO_4_ (pH 5.5), and 0.15 µM myo-[2-^3^H]-inositol (MP Biomedicals) (1 µCi/µl). An additional 200 µM concentration of unlabeled inositol (Sigma-Aldrich) was added to the reaction mixtures for competition assays. Equal volumes of the reaction and cell mixtures (60 µl each) were warmed to 30 °C and mixed for the uptake assay, which was performed for 10 min at 30 °C. As negative controls, mixtures were kept at 0 °C (on ice) during the 10-min incubation. Aliquots of 100 µl were removed and transferred onto prewetted Metricel filters (1.2-µm pore size) on a vacuum manifold. The filters were washed four times each with 2 ml of ice-cold water. The washed filters were removed and added to liquid scintillation vials for measurements on a PerkinElmer TRI-CARB 2900TR scintillation counter.

### Glucose uptake assay

Full-length cDNAs of the *ITR4*, *ITR5* and *ITR6* genes were amplified from *C. neoformans* KN99**a** total cDNA and were cloned into the yeast expression vector pTH74 to generate GFP fusion constructs, under the control of the *ADH1* promoter. *ITR4, ITR5* and *ITR6* expression plasmids were introduced into an *S. cerevisiae* strain EBY.VW1000 that lacks all 20 *HXT* transporters [29]. The expression of *Cryptococcus ITR4, ITR5* or *ITR6* in this yeast heterologous system was verified by both GFP localization and qRT-PCR using gene-specific primers. Yeast strains were tested for growth on different medium at 30 °C.

The *S. cerevisiae* control strain CEN.PK2.1C, the *S. cerevisiae hxt*Δ mutant strain EBY.VW1000, and EBY.VW1000 expressing empty vector, *ITR4:GFP*, *ITR5:GFP or ITR6:GFP* genes from *C. neoformans* were grown in YP containing 2% maltose liquid cultures overnight at 30 °C. Collected cells were washed with dH_2_O, resuspended in YP containing 2% maltose, and incubated for 2 h. Then cells were suspended in PBS at a final concentration of 2 × 10^8^ cells/ml for uptake assay. Each 100 μl cell suspension was mixed with 100 μl labeled glucose (^3^H-glucose) solution at room temperature. Samples (100 μl) were removed after 30 s, 1 min, 5 min, and 10 min, and mixed with 1 ml ice-cold water to stop the reactions. Cells were immediately collected on fiber filters, washed three times with 10 ml of ice-cold water, and transferred to scintillation vials for measurement.

### Detection of *ITR* gene expression using qRT-PCR

To test how the *ITRs* respond to the presence of environmental myo-inositol, both in vitro and in vivo, we measured the mRNA levels for *ITR* genes under different conditions via quantitative real-time PCR (qRT-PCR). Cultures of *C. neoformans* wild-type strain KN99**a** and its mutant strains were grown on YPD for 24 h at 30 °C. Collected cells were washed with dH_2_O and pellets were used for total RNA extraction. Total RNAs were extracted using Trizol reagents (Invitrogen) and purified with Qiagen RNeasy cleanup kit (Qiagen) following the manufacturer’s instructions. Purified RNAs were quantified using a Nanodrop instrument (Thermo Scientific). First-strand cDNAs were synthesized using a Superscript III cDNA synthesis kit (Invitrogen) following the instructions provided by the manufacturer. Expression levels of *ITRs* and *GAPDH* were analyzed with the comparative C_T_ method using Brilliant SYBR green QPCR reagents (Takara) as described previously [11,21].

### Protein-protein interaction assay using the split-ubiquitin system

The split-ubiquitin system was utilized to investigate the interaction between Itr4 and Itr1A, Itr3C and Itr3B as previously described [56]. Vectors and yeast strains were included in the DUALmembrane Kit 2 (Dualsystem Biotech, Switzerland). *ITR4* full-length and *ITR4*^*∆11-119*^ cDNA were cloned into yeast expression vector pCCW (the N-terminal half of the ubiquitin Cub protein was fused to the C-terminus of *ITR4* and *ITR4*^*∆11-119*^), and *ITR1A*, *ITR3C*, *ITR3B* full-length cDNA and *ITR4*^*∆11-119*^, *ITR4*^*Q388A, Q389A*^ cDNA were cloned into the pDL2Nx vector (the mutated C-terminal half of ubiquitin NubG protein was fused to the *ITR1A*, *ITR3C*, *ITR3B*, *ITR4, ITR4*^*∆11-119*^ and *ITR4*^*Q388A, Q389A*^ N-terminus). All cDNA sequences were confirmed by DNA sequencing. Cub and NubG fusion constructs were co-transformed into host yeast strain NMY32. Two constructs, pCCW-Alg5 and pAI-Alg5, and pCCW and pDL2Nx serve as positive and negative control. Interaction was determined by the growth of yeast transformants on medium lacking histidine or adenine, and also by measuring β-galactosidase enzyme activity assays using Chlorophenolred-β–D-galactopyranoside (CRPG; Calbiochem, San Diego, CA) as substrate.

### Fluorescent signal quantification and data analysis

Plasmids expressing *P*_*ACT1*_*-ITR4:mCherry* fusion protein, *P*_*ACT1*_*-ITR4*^*∆11-119*^*:mCherry* fusion protein and *P*_*ACT1*_*-ITR4*^*Q388A,Q389A*^*:mCherry* fusion protein were linearized and transformed into *itr4*Δ mutant to generate the *itr4*Δ *ITR4*^*OE*^*, itr4*Δ *ITR4*^*∆11-119*^ and *itr4Δ ITR4*^*Q388A, Q389A*^ strains. Strains were cultured in YPD overnight and were observed with a Nikon fluorescence microscope.

The percentage of mCherry signals localized within the cell (% cytosolocalized mCherry) was calculated in accordance with data analysis seen in Hankins and colleagues [60]. The outside of each cell was outlined using the freehand selection tool in ImageJ and then the total fluorescence of the cell was measured (Fluor_total_). The area just within the plasma membrane was then outlined to give the total internal fluorescence excluding the plasma membrane (Fluor_int_). The percentage of fluorescence localized within the cell was found by dividing Fluor_int_ by Fluor_total_.

### *Cryptococcus*-macrophage interaction assay

The *Cryptococcus-*macrophage interaction assay was done as previously described [52]. Macrophage-like J774 cells were cultured in DME medium with 10% heat-inactivated FBS at 37 °C with 5% CO_2_. J774 cells (5 × 10^4^) in 0.5 ml fresh DME medium were added into each well of a 48-well culture plate and incubated at 37 °C in 5% CO_2_ overnight. To activate macrophage cells, 50 units/ml gamma interferon (IFN-ϒ; Invitrogen) and 1 µg/ml lipopolysaccharide (LPS; Sigma) were added into each well. *C. neoformans* overnight cultures were washed with phosphate-buffered saline (PBS) twice and opsonized with 20% mouse complement. *Cryptococcus* cells (2 × 10^5^) were added into each well (yeast/J774 ratio, 4:1). To assess the phagocytosis rate, the cells were washed with PBS after a 1.5 h coincubation and fixed with methanol for 30 min. Giemsa stain was added to the wells at a 1:5 dilution, and the plates were incubated overnight at 4 °C. Cells were washed once with PBS and analyzed using an inverted microscope. To assess intracellular proliferation of *C. neoformans*, nonadherent extracellular yeast cells were removed by washing with fresh DME medium after a 4 h coincubation and cultures were incubated for another 0, 4, and 24 h. At indicated time points, the medium in each well was replaced with distilled water (dH_2_O) to lyse macrophage cells for 30 min at room temperature. The lysate was spread on YPD plates, and CFU were counted to determine intracellular proliferation.

### Human brain microvascular endothelial cells (HBMEC) transmigration assay

Primary isolates of HBMEC were cultured as previously described [61]. HBMEC were routinely grown in RPMI 1640 supplemented with 10% heat-inactivated fetal bovine serum, 10% Nu-serum, 2 mM glutamine, 1 mM sodium pyruvate, penicillin (100 units/ml), streptomycin (100 mg/ml), essential amino acids, and vitamins. The cells were incubated at 37 °C in a humidified incubator with 5% CO_2_. For all experiments, the same medium used for routine cell culture was used as the experimental medium.

The in vitro human BBB model was generated and used for fungal transmigration assays as previously described [62,63]. HBMEC were seeded on Transwell polycarbonate tissue culture inserts with a pore diameter of 8.0 μm (Corning Costar) and cultured until their TEER reached over 350 V/cm^2^, as measured by an Endohm volt/Ω meter in conjunction with an Endohm chamber (World Precision Instruments). The same medium used for cell culture was used as the experimental medium. Yeast cells were washed with phosphate-buffered saline (PBS) and resuspended in HBMEC culture medium. 10^5^ Cryptococcus cells were added to the top compartment and then incubated at 37 °C. At 3 and 24 h, the medium in bottom compartments was collected and immediately replaced with fresh medium. Fungal cell numbers in the collected medium were addressed by CFU counts to determine the number of transmigrated viable yeast cells. To determine the specificity of myo-inositol, the transmigration assay was performed in the presence of myo-inositol, D (+) galactose or D (+) maltose (1 mM each) in the bottom compartments prior to addition of Cryptococcus cells (10^5^) in the top compartment of Transwells. Results are presented as the total number in the bottom chamber. Each set was triplicated and repeated three times independently [13]. The statistical analysis of the data from our in vitro studies was done with a two-tailed Student *t* test. Statistical significance was determined at *P* < 0.001.

### RNA-seq quantification analysis

*C. neoformans* cells of the WT and *itr4∆* mutant strains were cultured overnight at 30 °C in YPD liquid medium and then inoculated in YNB + 1% Glucose + 1% Inositol condition for 24 h. The cells were then collected and total RNAs were extracted using Trizol reagents (Invitrogen) and purified with MACHEREY-NAGEL RNA purification kit following the manufacturer’s instructions. Purified RNAs were quantified using a Nanodrop instrument (Thermo Scientific). RNA-Seq analysis was performed by Novogene according to the company’s protocol (https://www.novogene.com/us-en). For the RNA-seq, cDNA library preparation, Illumina sequencing, and data analysis were performed by Novogene (Sacramento, CA). Sequencing was performed using Illumina NovoSeq 6000 to generate 150 bp paired-end reads. Approximately 50 million raw reads were generated. Adapter and low-quality reads or reads with uncertain nucleotides constitute more than 10 percent of either read (*N* > 10%), and reads with low-quality nucleotides (base quality less than 5) constitute more than 50 percent of the read, were removed. Clean reads were mapped to the annotated genome of *C. neoformans* H99 (NCBI:txid235443) using HISAT2

software (version 2.0.5). Differential expression analyses were conducted using the DESeq2 package (version 1.20.0) [64]. For each experimental condition included in the RNA-seq analysis, we performed three independent biological replicates. Significance was determined using multiple unpaired *T* tests with two-stage linear step-up.

### Murine infection

Survival curves of infected mice in a *Cryptococcus* murine inhalation model as previously described [21,25]. *Cryptococcus* strains were grown at 30 °C overnight and cultures were washed twice with 1× phosphate-buffered saline (PBS) by centrifugation and resuspended at a final concentration of 2 × 10^5^ CFU/ml. Groups of 10 female C57BL/6 mice (Jackson Laboratory, ME) were used for each infection. For the intranasal inhalation model, mice were intranasally infected with 10^4^ yeast cells of each strain in 50 µl PBS as previously described. For the intravenous injection model, 5 × 10^4^ yeast cells in 100µl volume for each strain were inoculated via tail vein injection. Over the course of the experiments, animals that appeared moribund or in pain were sacrificed by CO_2_ inhalation. Survival data from the murine experiments were statistically analyzed between paired groups using the log-rank test and the PRISM program 6.0 (GraphPad Software) (*P* values of <0.001 were considered significant).

### Isolation and measurement of yeast cells from mouse BALF

Yeast strains were grown at 30 °C overnight and cultures were washed twice with PBS buffer and resuspended to a final concentration of 2 × 10^7^ cells/mL. Groups of 5 female C57BL/6 mice were intranasally infected with 1 × 10^6^ yeast cells of each strain as previously described [65,66]. BALF samples were harvested at day 3 after inoculation. BALF was collected in 3 ml of 1× PBS buffer using a catheter inserted into the trachea of animal post-euthanasia, and airway-infiltrating cells were lavaged with ∼0.7 ml of 1× PBS at one time to a total volume of 3 ml. Cells were spun down and host cells were lysed by adding sterile ddH_2_O and incubating for 45 min at room temperature. *Cryptococcal* cells were examined under microscope. Capsule sizes and cell body sizes of more than 500 cells were measured and calculated.

### Histopathology and immune response analysis for infected organs

Infected animals were sacrificed at the end of the experiment according to the Rutgers University IACUC-approved animal protocol. To compare the fungal burdens and host inflammatory responses, the lungs, brains, and spleen were dissected and fixed in 10% formalin solution for section preparation at Rutgers University Histology Core Facility. Tissue slides were treated either with hematoxylin and eosin (H&E) staining for bronchus-associated lymphoid tissue, or with Grocott’s methenamine silver (GMS) staining for fungal morphology observation in vivo. Infected lungs, brains, and spleens were also isolated and homogenized (Ultra-Trra T8, IKA) in 3 ml cold 1× PBS buffer for 1 min for each type of organ. The tissue suspensions were serially diluted and plated onto YPD agar medium with ampicillin and chloramphenicol, and colonies were counted after 3 days of incubation at 30 °C.

Lung tissue total RNAs were extracted using Trizol reagents (Invitrogen) and purified with a RNeasy cleanup kit (Qiagen) following the manufacturer’s instructions. Purified RNAs were quantified using a Nanodrop instrument (Thermos Scientific). First-strand cDNAs were synthesized using a Superscript III cDNA synthesis kit (Invitrogen) following the instructions provided by the manufacturer. Expression levels of Cytokines and *ACTIN* were analyzed with the comparative C_T_ method using Brilliant SYBR green QPCR reagents (Takara) as described previously. Relative mRNA levels were determined by quantitative reverse transcription qRT-PCR. Gene expression relative to that of a naive sample was calculated by the ΔΔ*CT* method.

### In silico protein structure analysis

Structural modeling and ligand docking: The three-dimensional structures of Itr4 monomer and several dimers were predicted using AlphaFold2 in monomer and multimer modes, respectively [30]. AlphaFold2 was accessed through the COSMIC2 portal. The resulting PDB files were transferred to the Rutgers University Amarel high-performance compute cluster for analysis. The structures were assessed in Molecular Operating Environment (MOE) version 2024.06 and the structures with the fewest clashing residues were prepared in MOE. The Amber:EHT force field was used for all operations within MOE. For docking, the Itr4 monomer and the energy-minimized myo-inositol were analyzed within MOE. The residues within the transmembrane helices were saved as sets and labeled TM1-12 according to their location. A dummy atom was created and residues within 4.5 Å were selected and the ligand-receptor pair with the lowest energy was selected. The resulting pose was captured as an instance within a MOE storyboard, imaged, and the ligand interaction diagram was reported. This storyboard instance was the starting point for subsequent virtual mutations.

Digital Alanine scan: The protein builder tool was used within MOE to mutate Q388 and Q389 to alanine as monosubstituted and disubstituted models. The protein builder minimization tool was used to minimize the energy of the selected residues that were substituted. A molecular surface representing the local electrostatic environment was produced using receptor atoms within 4.5 Å of ligand atoms. The resulting mutant structure was saved as a new storyboard instance and the primary MOE viewer was reset to the original storyboard instance for a subsequent mutation.

### Molecular dynamics simulations

MD simulations were performed for the wild-type Itr4/inositol complex and the Itr4Q388A/Q389A/inositol complex. The initial binding poses of inositol were obtained from molecular docking. The prepared protein–ligand complexes were embedded in a DPPC (325K) lipid bilayer using the Membrane Builder module in Desmond (D. E. Shaw Research, NY; Maestro-Desmond Interoperability Tools, Schrödinger, NY, 2023). The place of membrane was further corrected based on the TMHMM results. The membrane-protein systems were then solvated with the SPC water model, and an orthorhombic simulation box was constructed with a minimum buffer distance of 10 Å * 10 Å * 21 Å in *x*, *y*, *z* directions. For MD workflow, the simulation time was set as 200 ns with recording intervals was set as 20 ps resulting in 10,000 frames for further analysis. Other parameters were set as default. Trajectory visualization and structural analyses were performed using the Maestro graphical user interface (GUI). Protein and ligand root-mean-square deviations (RMSDs) were calculated throughout the simulation. Protein–ligand interaction frequencies were analyzed using the Simulation Interaction Diagram (SID) tool implemented in the Desmond module to evaluate the stability of inositol binding and the contribution of individual residues to ligand recognition.

## Supporting information

S1 Table *Cryptococcus neoformans* strains used in this study. (DOCX)

S2 Table *Saccharomyces cerevisiae* strains used in this study. (DOCX)

S3 Table Primers used in this study. (DOCX)

S4 Table Top 50 genes down-regulated in the *itr4∆* mutant cells. (DOCX)

S5 Table Top 50 genes up-regulated in the *itr4∆* mutant cells. (DOCX)

S6 Table Differentially expressed genes involved in glucose metabolism. (DOCX)

S7 Table SEC-MALS analysis of shed extracellular polysaccharide (EPS) from different strains. (DOCX)

S8 Table Expression profile of genes involved in inositol function and glucose signaling. (XLSX)

S1 Fig Phenotypic analysis of group II *ITRs.* **(A)** Cultures were grown overnight in YPD and diluted to an optical density at 600 nm (OD600) of 2.0. Tenfold serial dilutions were made in dH2O, and 5 ul of each was plated. The plates were grown for 2 days at 30 and 37 °C. **(B)** Melanin detection assay. Color development by *itr4∆*, *itr5∆*, and *itr6∆* single mutants and their triple mutants (*itr4∆ itr5∆ itr6∆*) and wild-type KN99α strains was evaluated after cultures were grown on Niger Seed plate for 48 h at 30 and 37 °C. **(C)** Cultures were grown overnight in YPD and diluted to an OD600 of 2.0. Serial dilutions and spotting assays were made, and the plates were grown for 2 days at 30 and 37 °C for different stress and inhibitor conditions. Wild-type and mutant strains are indicated on the left and the conditions at the top.
(TIF)

S2 Fig Itr4 N-terminal tail is required for maintaining the Itr4 function. **(A)** Expression of genes involved in inositol transport, glucose metabolism in the *itr4Δ* mutant and *itr4Δ+ITR4*^*Q388A, Q389A*^. Relative transcript levels of the indicated genes were determined by quantitative qRT-PCR in wild-type, the *itr4Δ* mutant and *itr4Δ+ITR4*^*Q388A, Q389A*^ strains grown under 1% glucose + 1% inositol condition. Gene expression was normalized to the internal control and is presented relative to wild-type KN99**a**. Each dot represents an independent biological replicate, and bars indicate the mean ± SD. Statistical significance was determined using an unpaired two-tailed Student *t* test. “****”, *P* < 0.0001; “***”, *P* < 0.001; ns, not significant. **(B)** Detection of potential interactions between Itr4, Itr3B, Itr4^∆11-119^ and Itr4^Q388A, Q389A^ in a yeast split-ubiquitin system. Two constructs, pCCW-Alg5 and pAI-Alg5, and pCCW and pDL2Nx serve as positive and negative control. Interaction was determined by the growth of yeast transformants on medium lacking histidine or adenine, and also by measuring β-galactosidase enzyme activities to verify the interaction. **(C)** Predicted Itr4 homodimer self-interactions. Depicts interactions of chain 1 N′ residues 97–115 with chain 2 C′ residues 629–630 was shown on left (L), and depicts interactions of chain 1 N′ residues 112–120 with chain 2 C′ residues 605–606 wa shown on right (R). The data underlying this figure can be found in the supplemental information file S2 Data.
(TIF)

S3 Fig Itr4 protein structure modeling and predicted sugar binding sites. **(A)** Itr4 protein structure model predicted by AlphaFold2. Green color represents the Itr4 protein ribbon. Red region represents the first 120 amino acids of the N-terminal tail located within the cytoplasm. Blue region represents intracellular loops in Itr4. **(B)** Schematic of mutation sites in Itr4 protein. The N-terminal region between residues 11 and 119 amino acids was deleted, while residues Q388, Q389 and F497 were replaced by alanine (A). **(C)** Results from the virtual alanine mutation scan from the protein builder tool in Molecular Operation Environment (MOE). Receptor surface maps within 4.5Å of the ligand, shown as line diagrams, represent the shapes of the binding pockets in the wild-type (WT) and mutated forms of the protein. Red lines represent a negatively charged environment, blue lines represent a positively charged environment, and white lines represent neutral environments. The Q388A mutation had a significantly larger impact on the size of the binding pocket shape and volume than the Q389A mutation. Ligand interaction diagrams represent the non-covalent interactions between the ligand and receptor. Potential energy calculations computed did not account for hydration energies and may not fully capture the reality of the living cell.
(TIF)

S4 Fig Heterologous expression and uptake assays of *ITR4* in *Saccharomyces* strains lacking inositol transporter. **(A)** Localization of *ITR4, ITR4*^*∆11-119*^*, ITR4*^*Q388A, Q389A*^ and *ITR4*^*F497A*^ in *S. cerevisiae* was determined by overexpressing of their GFP fusion protein in *Sc itr1 itr2* mutant lacking two inositol transporters. **(B)** Inositol uptake analysis of *Cryptococcus ITR4, ITR4*^*∆11-119*^*, ITR4*^*Q388A, Q389A*^ and *ITR4*^*F497A*^ expressed in a yeast heterologous system. Yeast cells were mixed with ^3^H-labeled inositol and incubated at 30 °C for 10 min. Error bars indicate standard deviations of data from at least three independent experiments. *Sc*, *S*. *cerevisiae*. The data underlying this figure can be found in the supplemental information file S2 Data.
(TIF)

S5 Fig Western blot analysis to detect the GFP fusion protein expression of itr4 and its muant alleles. **(A)** western blot analysis to examine the expression and stability of Itr4^Δ11–119^, Itr4 and the Itr4^Q388A, Q389A^ GFP fusion proteins in *S. cerevisiae* with a GFP antibody. **(B)** western blot analysis to examine the expression and stability of Itr4^Δ11–119^, Itr4^OE^ and the Itr4^Q388A, Q389A^ mCherry fusion proteins in *C. neoformans* with a mCherry antibody.
(TIF)

S6 Fig Structural characterization of GXM polymers. **(A)** SEC-MALS analysis of extracellular polysaccharide (EPS) from KN99**a**, *itr4∆,* and *ITR4*^*Q388,389A*^ strains in glucose growth conditions. **(B)** 1D [^1^H] NMR analysis of EPS from all 3 strains. **(C)** Characterizing GXM motif expression and GXM O-acetylation.
(TIF)

S7 Fig Itr4 is required for fungal virulence in in vivo murine models of systemic cryptococcosis. **(A)** Expression profile of cytokine genes and genes involved in macrophage polarization was examined by qRT-PCR in mouse lung at day 7 after infection. Samples from naïve mice were analyzed as a control. Relative expression levels of indicated genes were calculated using the ΔΔCt method and normalized to Actin gene. **(B and C)** Histological examination of lung apex and parenchyma sections. The tissue sections were fixed and stained with H&E and GMS. Fungal cells appear dark brown or black with GMS staining. Yeast cells are highlighted with black arrow. Bar, 20 µm. The data underlying this figure can be found in the supplemental information file S2 Data.
(TIF)

S8 Fig Itr4 s required for fungal dissemination to brain to cause brain infection in an in vivo murine infection model. **(A)** End-point fungal burdens in the lungs, brains, spleens of animals infected with indicated strains in a murine intranasal inhalation model of systemic cryptococcosis. **(B)** End-point fungal burdens in the lungs, brains, spleens of animals infected with indicated strains in a murine intravenous injection infection model of systemic cryptococcosis. Each symbol represents one mouse. Bars represent the mean values ± standard errors of the means. “****”, *P <* 0.0001; “***”, *P* *<* 0.001 “**”, *P <* 0.01; “*”, *P* *<* 0.05 (determined by One-way ANOVA nonparametric test for multiple comparisons). **(C and D)** Histological examination of brain sections. The brain sections were fixed and stained with H&E and GMS. Fungal cells appear dark brown or black with GMS staining. Yeast cells are highlighted with black arrow. Bar, 20 µm. The data underlying this figure can be found in the supplemental information file S2 Data.
(TIF)

S1 Data Data underlying all diagrams of the main manuscript. (XLSX)

S2 Data Data underlying all diagrams of the supporting information figures. (XLSX)

S1 Raw Images Data supplement for Western Blot full gel images. (PDF)

## Abbreviations

BALF: bronchoalveolar lavage fluid
BBB: blood–brain barrier
BP: Biological Process
CC: Cellular Component
CCR: carbon catabolite repression
CFW: calcofluor white
CNS: central nervous system
COAT: Cryptococcal Optimal Antiretroviral Therapy Timing
CSF: cerebral spinal fluid
CTR: C-terminal region
DEG: differentially expressed genes
DME: Dulbecco modified Eagles
EPS: extracellular polysaccharides
GMS: Grocott’s methenamine silver
GUI: graphical user interface
GWAS: Genome-wide association studies
HBMEC: Human brain microvascular endothelial cells
IACUC: Institutional Animal Care and Use Committee
IPA: Ingenuity Pathway Analysis
ITRs: inositol transporters
MD: molecular dynamics
MF: Molecular Function
MIOs: member inositol oxygenases
MITS: myo-inositol transporters
MOE: Molecular Operating Environment
MS: Murashige and Skoog
PBS: phosphate-buffered saline
PHS: Public Health Services
RMSDs: root-mean-square deviations
SID: Simulation Interaction Diagram
TEER: the Transendothelial Electrical Resistance
WT: wild type
YPD: yeast extract-peptone-dextrose
