# Supplementary material for: An inositol receptor Itr4 coordinates carbon catabolite regulation and virulence in Cryptococcus neoformans
Source: PLoS Biol. 2026 Oct 13;24(10):e3003709. doi: 10.1371/journal.pbio.3003709 (PMC13686456; doi:10.1371/journal.pbio.3003709)
Supplement: Supplementary file 2 [file pbio.3003709.s001.docx]

S1 Table. *Cryptococcus neoformans* strains used in this study

| ***C. neoformans* strains** | **Genotype** | **Source/reference** |
| --- | --- | --- |
| H99 | *MAT*α | [1] |
| KN99**a** | *MAT***a** | [2] |
| KN99α | *MAT*α | [2] |
| CUX869 | *MATα itr6::NAT* | UCSF |
| CUX870 | *MATα itr5::NAT* | UCSF |
| CUX871 | *MATα itr4::NAT* | UCSF |
| CUX1029 | *MAT***a** *itr4::NEO* | This study |
| CUX1030 | *MATα itr4::NAT ITR4-NEO* | This study |
| CUX1031 | *MATα itr4::NEO itr6::NAT* | This study |
| CUX1071 | *MATα itr4::NEO itr5::NAT itr6::NAT* | This study |
| CUX1072 | *MAT***a** *itr4::NEO itr5::NAT itr6::NAT* | This study |
| CUX1121 | *MAT***a** *itr4::NEO pITR4-ITR4:mCherry-NAT* | This study |
| CUX1122 | *MAT***a** *itr4::NEO pACT-ITR4:mCherry-NAT* | This study |
| CUX1123 | *MAT***a** *itr4::NEO pACT-ITR4 ^Δ11-119^:mCherry-NAT* | This study |
| CUX1124 | *MAT***a** *itr4::NEO pACT-ITR4 ^Q388A Q389A^:mCherry-NAT* | This study |
| CUX1125 | *MAT***a** *itr4::NEO pACT-ITR^F497A^:mCherry-NAT* | This study |

**References**

1. Perfect JR, Schell WA, Rinaldi MG. Uncommon invasive fungal pathogens in the acquired immunodeficiency syndrome. J Med Vet Mycol. 1993;31(2):175-9. doi: 10.1080/02681219380000211. PMID: 8509954.
2. Nielsen K, Cox GM, Wang P, Toffaletti DL, Perfect JR, Heitman J. Sexual cycle of Cryptococcus neoformans var. grubii and virulence of congenic a and alpha isolates. Infect Immun. 2003 Sep;71(9):4831-41. doi: 10.1128/IAI.71.9.4831-4841.2003. PMID: 12933823; PMCID: PMC187335.
