# Supplementary material for: An inositol receptor Itr4 coordinates carbon catabolite regulation and virulence in Cryptococcus neoformans
Source: PLoS Biol. 2026 Oct 13;24(10):e3003709. doi: 10.1371/journal.pbio.3003709 (PMC13686456; doi:10.1371/journal.pbio.3003709)
Supplement: Supplementary file 3 [file pbio.3003709.s002.docx]

S2 Table. *Saccharomyces cerevisiae* strains used in this study

| **Strains** | **Genotype** | **Source/reference** |
| --- | --- | --- |
| BY4741 | *MAT A his3∆1, leu2∆0, met15∆0, ura3∆0* | ATCC yeast deletion collection |
| BY4742 | *MAT Alpha his3∆1, leu2∆0, ura3∆0* | ATCC yeast deletion collection |
| CEN.PK2-1C | *leu2-3,112 ura3-52 trp1-289 his3∆1 MAL2-8c SUC2 hxt7∆* | [29] |
| EBY.VW1000 | *CEN.PK2-1C hxt13∆::loxP hxt15∆::loxP hxt16∆::loxP hxt14∆::loxP hxt12∆::loxP hxt9∆::loxP hxt11∆::loxP hxt10∆::loxP hxt8∆::loxP hxt514∆::loxP hxt2∆::loxP* | [29] |
| MNY32 | *MATa his3∆200 trp1-901 leu2-3,112 ade2 LYS2::(lexAop)4-HIS3 URA3::(lexAop)8-lacZ ade2::(lexAop)8-ADE2 GAL4* | Dualsystem |
| YUX13 | *MAT Alpha, his3∆1, leu2∆0, ura3∆0, itr1∆, itr2∆* | Previous study |
| YUX83 | *scrgt2::NAT scsnf3::NEO P_ADH1_-GFP:HXS1* | Previous study |
| YUX116 | *EBY.VW1000 P_ADH1_-GFP:ITR6* | This study |
| YUX117 | *EBY.VW1000 P_ADH1_-GFP:ITR5* | This study |
| YUX118 | *EBY.VW1000 P_ADH1_-GFP:ITR4* | This study |
| YUX120 | *EBY.VW1000 P_ADH1_-GFP* | This study |
| YUX120 | *Sc itr1 itr2 P_ADH1_-GFP* | This study |
| YUX121 | *Sc itr1itr2 P_ADH1_-GFP:ITR1A* | This study |
| YUX122 | *Sc itr1itr2 P_ADH1_-GFP:ITR3C* | This study |
| YUX123 | *Sc itr1itr2 P_ADH1_-GFP:ITR4* | This study |
| YUX125 | *Sc itr1itr2 P_ADH1_-GFP:ITR5* | This study |
| YUX127 | *Sc itr1itr2 P_ADH1_-GFP:ITR6* | This study |
| YUX133 | *Sc itr1itr2 P_ADH1_-GFP:ITR4 ^Δ11-119^* | This study |
| YUX134 | *Sc itr1itr2 P_ADH1_-GFP:ITR4 ^Q388A Q389A^* | This study |
| YUX135 | *Sc itr1itr2 P_ADH1_-GFP:ITR4 ^Q497A^* | This study |
| YUX136 | *NMY32 pCCW-ITR4 pDL2Nx-ITR4* | This study |
| YUX137 | *NMY32 pCCW-ITR4 pDL2Nx-ITR1A* | This study |
| YUX138 | *NMY32 pCCW-ITR4 pDL2Nx-ITR3C* | This study |
| YUX139 | *NMY32 pCCW-ITR4 pDL2Nx-ITR3B* | This study |
| YUX140 | *NMY32 pCCW pDL2Nx* | This study |
| YUX141  YUX200  YUX201  YUX202  YUX203  YUX204 | *NMY32 pCCW-Alg5 pAI-Alg5*  *NMY32 pCCW-ITR4 pDL2Nx-ITR4* *^∆11-119^*  *NMY32 pCCW-ITR4 pDL2Nx-ITR4* ^Q388A Q389A^  *NMY32 pCCW-ITR4^∆11-119^ pDL2Nx-ITR3B*  *NMY32 pCCW-ITR4^∆11-119^ pDL2Nx-ITR4*  *NMY32 pCCW-ITR4^∆11-119^ pDL2Nx-ITR4* *^∆11-119^* | This study  This study  This study  This study  This study  This study |
