# Supplementary material for: An inositol receptor Itr4 coordinates carbon catabolite regulation and virulence in Cryptococcus neoformans
Source: PLoS Biol. 2026 Oct 13;24(10):e3003709. doi: 10.1371/journal.pbio.3003709 (PMC13686456; doi:10.1371/journal.pbio.3003709)
Supplement: Supplementary file 4 [file pbio.3003709.s003.docx]

S3 Table. Primers used in this study

| **Primer name** | **Sequence (5’-3’)** | **Description** |
| --- | --- | --- |
| CX5 | GTAAAACGACGGCCAG | M13F |
| CX6 | CAGGAAACAGCTATGAC | M13R |
| JH8994 | TGTGGATGCTGGCGGAGGATA | JH8994 |
| CX249 | GAGGCTATTCGGCTATGACTGG | NEO split F |
| CX2281 | AGGCTGGCAAATCAAGCGTG | ITR4 F1 |
| CX2282 | CTGGCCGTCGTTTTACCGTGGTGATGGTGGTGCTCGAG | ITR4 R1 |
| CX2283 | GTCATAGCTGTTTCCTGACGGGGAGAAAGGCGTAGAGG | ITR4 F2 |
| CX2284 | CAACTTCCCAATACATCATG | ITR4 R2 |
| CX1010 | CTGAGATTGCTCCCGCTAGG | ITR4 F3 |
| CX1011 | AATGAACACCACCCAAGCCA | ITR4 R3 |
| CX1009 | TGCAGTTTACATTTCCAATCGTC | ITR4 F4 |
| CX2285 | CAGGTATTTGACTAGTCTGC | ITR4 R4 |
| CX1419 | TACCGAGCTCGGATCCGACTCTCACGTCGTTGTATAC | pJAF1-ITR4 F |
| CX1420 | CGTTACTAGTGGATCCTCCAAGCACCCATCACTACATG | pJAF1-ITR4 R |
| CX1800 | CAACATGTCTGGATCCATGTCCACGCTTGACTACAAG | pCXU200-ITR4 F |
| CX1801 | TAGAACTAGTGGATCCAGCCTTCGACCGCTTTTCATT | pCXU200-ITR4 R |
| CX2274 | GAATTCCCGGGGATCCAGATGTCCACGCTTGACTACAAGGCCGACGCCGGTCCTTGGATGCTCTGGTACTCG | pTH74-ITR4 N-deletion in-fusion F |
| CX2275 | GCTTCTACTGTTATGCTTGCTGCGGCGATGTGCGGTATCAACATCATC | pTH74-ITR4 388 389 Mutation F |
| CX2276 | GATGATGTTGATACCGCACATCGCCGCAGCAAGCATAACAGTAGAAGC | pTH74 ITR4 388 389 Mutation R |
| CX2279 | CTCGGTGAAGGTCCCGTCGCTGCCCAGTACTCTGCAGAAGTCTTC | pTH74 ITR4 497 Mutation F |
| CX2280 | GAAGACTTCTGCAGAGTACTGGGCAGCGACGGGACCTTCACCGAG | pTH74 ITR4 497 Mutation R |
| CX2285 | CAGGTATTTGACTAGTCTGC | ITR4 R4 reverse |
| CX2286 | ATCATTGCCGCAAGGGACTAC | ITR4 F8 for sequencing |
| CX2287 | CCGACGATCCATTCGTCTCGG | ITR4 R8 for sequencing |
| CX2288 | CAACATGTCTGGATCCATGTCCACGCTTGACTACAAGGCCGACGCCGGTCCTTGGATGCTCTGG | pCXU200-ITR4 N-deletion in-fusion F1 |
| CX2289 | TAGAACTAGTGGATCCAGCCTTCGACCGCTTTTCA | pCXU200-ITR4 N-deletion in-fusion R |
| CX2290 | CAACATGTCTGGATCCATGTCCACGCTTGACTACAAGG | pCXU200-ITR4 N-deletion in-fusion F2 |
| CX2301 | ACGGTATCGATAAGCTTGTCAATACGACCAAAATTGC | pCXU200-ITR4 Native Promoter in-fusion F |
| CX2302 | TGCAGCCCGGGGGATCCGTCAATACGACCAAAATTGC | pCXU199-ITR4 Native Promoter in-fusion F |
| CX2316 | ACGGTATCGATAAGCTTGGCAGATACGATATGTTGGCG | pCXU199-H4 in-fusion F |
| CX2317 | CTAGAACTAGTGGATCCGGTGATAGATGTGTTGTGGTGT | pCXU199-H4 in-fusion R |
| CX2318 | ATCTATCACCGGATCCATGTCCACGCTTGACTACAAG | pCXU459-ITR4 infusion F |
| CX2319 | TAGAACTAGTGGATCCAGCCTTCGACCGCTTTTCATT | pCXU459-ITR4 infusion R |
| CX2320 | ATCTATCACCGGATCCATGTCCACGCTTGACTACAAGGCCGACGCCGGTCCTTGGATGCTCTGG | pCXU459-ITR4 N-deletion infusion F |
| CX2363 | CAAGACTAAAGGACGAGCAGAG | CNAG_05665 QPCR F |
| CX2364 | CACGGGAAATGGAGGATGTAG | CNAG_05665 QPCR R |
| CX2365 | CACCGATGGTTCATCCCTATAC | CNAG_05664 QPCR F |
| CX2366 | GGACATAGCGTATCCCTTCTTC | CNAG_05664 QPCR R |
| CX2367 | GCTGGCATTACGAAGGAAGA | CNAG_05668 QPCR F |
| CX2368 | CCAAGGAGTTGAGGTCGTTAAT | CNAG_05668 QPCR R |
| CX2369 | TACCATTTGGAAAGCCCTCTAC | CNAG_05666 QPCR F |
| CX2370 | GTGAAGCTGCTCGACACTAATA | CNAG_05666 QPCR R |
| CX2371 | AGACAGAGACAGAGACGATGAG | CNAG_05663 QPCR F |
| CX2372  CX2504  CX2519  CX2530 | CCGACAGAAACAGAACCAGTAG  GCAAACACAAATACACACACTAATCTAGACGGCCATTACGGCCATGTCCACGCTTGACTACAAG  AATTCCTGCAGATATACCCATGGAGGCCTTTGGCCGAGGCGGCCAGCCTTCGACCGCTTTTCATT  GGCCTCTCGAGAATTCATGTCGGACGAGAAGAACTATG | CNAG_05663 QPCR R  pCCW-ITR4 in-fusion F  pCCW-ITR4 in-fusion R  pDL2Nx-ITR1A in-fusion F |
| CX2531 | GCTTGATATCGAATTCAACTTTGTTGAGCTGGTCATCAG | pDL2Nx-ITR1A in-fusion R |
| CX2532 | GGCCTCTCGAGAATTCATGAGCAGAACACCGTCCAG | pDL2Nx-ITR3C in-fusion F |
| CX2533 | GCTTGATATCGAATTCTCTTGAGATCGGAAACCTCT | pDL2Nx-ITR3C in-fusion R |
| CX2534 | GGCCTCTCGAGAATTCATGGCCATTTTGATATCTGATG | pDL2Nx-ITR3B in-fusion F |
| CX2535 | GCTTGATATCGAATTCAGTTGCGGTTTTAACCAACAC | pDL2Nx-ITR3B in-fusion R |
| CX2536 | GGCCTCTCGAGAATTCATGTCCACGCTTGACTACAAG | pDL2Nx-ITR4 in-fusion F |
| CX2537 | GCTTGATATCGAATTCAGCCTTCGACCGCTTTTCATT | pDL2Nx-ITR4 in-fusion R |
