# Supplementary material for: An inositol receptor Itr4 coordinates carbon catabolite regulation and virulence in Cryptococcus neoformans
Source: PLoS Biol. 2026 Oct 13;24(10):e3003709. doi: 10.1371/journal.pbio.3003709 (PMC13686456; doi:10.1371/journal.pbio.3003709)
Supplement: Supplementary file 5 [file pbio.3003709.s004.docx]

S4 Table. Top 50 genes down-regulated in the *itr4∆* mutant cells

| **Gene_ID** | **Name** | **Function description** | **ITR4_GI1** | **ITR4_GI2** | **ITR4_GI3** | **WT_GI1** | **WT_GI2** | **WT_GI3** | **log2FoldChange** | **pvalue** | **gene_chr** |
| --- | --- | --- | --- | --- | --- | --- | --- | --- | --- | --- | --- |
| CNAG_05662 | ITR4 | Inositol sensor | 0 | 0 | 0 | 14.6911605 | 14.6695604 | 14.6682221 | -17.188133 | 3.8823E-48 | 14 |
| CNAG_05665 |  | hypothetical protein | 0 | 0 | 0 | 13.1269964 | 13.1507405 | 13.108171 | -15.640628 | 3.7887E-40 | 14 |
| CNAG_05664 | BAT1 | Branched-chain-amino-acid transaminase | 0 | 0 | 0 | 11.0064427 | 10.9361692 | 11.0938602 | -13.524173 | 2.1888E-30 | 14 |
| CNAG_05667 | ITR3B | myo-inositol transporter | 0 | 0 | 0 | 10.2787153 | 10.4248922 | 10.320484 | -12.854625 | 1.3936E-27 | 14 |
| CNAG_13204 |  | ncRNA gene | 0 | 0 | 0 | 9.56542867 | 9.48162926 | 9.59016088 | -12.057371 | 2.0471E-24 | 14 |
| CNAG_13206 |  | ncRNA gene | 0 | 0 | 0 | 8.51072278 | 8.60299744 | 8.47340863 | -11.043078 | 1.2555E-20 | 14 |
| CNAG_07892 |  | hypothetical protein | 0.67614713 | -0.0109664 | 1.71253289 | 9.89538663 | 9.91572509 | 9.72859099 | -8.9323148 | 1.4584E-50 | 14 |
| CNAG_03772 | HXS1 | high-affinity glucose transporter | 7.9097668 | 7.96631351 | 8.01021344 | 15.2037046 | 15.2215176 | 15.1451293 | -7.2314826 | 0 | 2 |
| CNAG_05663 | SCW1 | cell wall integrity protein | 4.72054125 | 5.07649643 | 4.12757039 | 11.5089793 | 11.4248922 | 11.5514111 | -6.7881228 | 0 | 14 |
| CNAG_07893 |  | hypothetical protein | 1.67614713 | 1.98903359 | 1.12757039 | 7.57212332 | 7.66850739 | 7.36436587 | -5.890384 | 8.9671E-33 | 14 |
| CNAG_04837 | MLN1 | bHLH family transcription factor | 5.97992788 | 5.60374343 | 5.58700201 | 10.6140866 | 10.5618515 | 10.5527197 | -4.8223084 | 7.694E-275 | 10 |
| CNAG_00827 |  | ribose 5-phosphate isomerase | 4.53412812 | 4.38135101 | 4.8824579 | 9.31309085 | 9.51388019 | 9.47892564 | -4.8341272 | 1.223E-132 | 1 |
| CNAG_02781 |  | dihydrodipicolinate synthetase | 8.26860416 | 7.9432299 | 8.32232725 | 12.9868477 | 12.9431522 | 12.934096 | -4.7666905 | 0 | 3 |
| CNAG_04142 |  | tartrate transporter | 10.3626477 | 10.3064462 | 10.620425 | 15.2073712 | 15.2532592 | 15.1396973 | -4.7664275 | 0 | 9 |
| CNAG_01680 |  | 3-methylcrotonyl-CoA carboxylase alpha subunit | 3.37658685 | 3.15895859 | 2.12757039 | 8.07462366 | 8.28018723 | 7.74012338 | -5.028319 | 8.4682E-51 | 11 |
| CNAG_13202 |  | ncRNA gene | -0.3238529 | -0.0109664 | 0.12757039 | 4.62459074 | 4.45060236 | 4.14594235 | -4.5067101 | 4.0035E-06 | 14 |
| CNAG_02044 |  | hypothetical protein | 8.11077536 | 8.17390893 | 8.16648938 | 12.4931929 | 12.5105933 | 12.6012696 | -4.3864432 | 0 | 6 |
| CNAG_02118 |  | hypothetical protein | 6.33435861 | 6.34658559 | 6.27731751 | 10.6018707 | 10.7678183 | 10.546165 | -4.3216051 | 1.032E-265 | 6 |
| CNAG_05303 | ICL1 | isocitrate lyase | 8.02044304 | 8.05512278 | 7.77862209 | 12.1055252 | 12.07138 | 12.2202897 | -4.1710267 | 0 | 4 |
| CNAG_00269 |  | Sorbitol dehydrogenase | 3.76360997 | 3.15895859 | 3.71253289 | 7.64378431 | 7.66850739 | 8.00009149 | -4.195324 | 4.1053E-46 | 1 |
| CNAG_03666 |  | acyl-CoA dehydrogenase | 5.48350205 | 5.54362244 | 6.21503324 | 10.2030982 | 10.3595823 | 10.1090607 | -4.4674524 | 3.007E-193 | 2 |
| CNAG_04025 |  | transaldolase | 9.95807715 | 9.81717007 | 10.0583077 | 13.9759244 | 13.9764636 | 13.8938771 | -4.0024236 | 0 | 2 |
| CNAG_00598 |  | nicotinamide mononucleotide permease | 6.78467158 | 6.71695404 | 6.72748324 | 10.6595862 | 10.6685074 | 10.5408997 | -3.8799206 | 7.59E-270 | 1 |
| CNAG_06551 |  | Carnitine O-acetyltransferase | 8.8634992 | 8.7472568 | 8.78220642 | 12.5975705 | 12.5932644 | 12.5927076 | -3.7933712 | 0 | 7 |
| CNAG_02045 |  | acetoacetate-CoA ligase | 7.92407464 | 7.90789682 | 7.54542291 | 11.5076704 | 11.5096529 | 11.536938 | -3.7060574 | 0 | 6 |
| CNAG_00826 |  | dihydroxyacetone kinase | 5.86597169 | 6.03342771 | 5.58700201 | 9.52113939 | 9.55639702 | 9.55141114 | -3.6958749 | 3.613E-142 | 1 |
| CNAG_06389 |  | hypothetical protein | 8.55573038 | 8.39842452 | 8.42219114 | 12.1366257 | 12.1343598 | 12.1953721 | -3.690482 | 0 | 13 |
| CNAG_07862 |  | fumarate reductase | 7.74760949 | 7.75721791 | 7.77862209 | 11.3339208 | 11.3951553 | 11.4184824 | -3.6219954 | 0 | 10 |
| CNAG_01653 | CIG1 | cytokine inducing-glycoprotein | 11.7731916 | 11.7028497 | 11.9695278 | 15.4203695 | 15.4404596 | 15.3589998 | -3.5881003 | 0 | 11 |
| CNAG_12392 |  | ncRNA gene | 11.5200682 | 11.3705765 | 11.6053287 | 15.0228764 | 15.1721474 | 15.0620515 | -3.5854481 | 0 | 4 |
| CNAG_06932 |  | Sugar transporter | 6.10241188 | 6.1183166 | 5.77142658 | 9.61327543 | 9.51762745 | 9.38508185 | -3.495852 | 1.65E-126 | 3 |
| CNAG_05340 |  | Monosaccharide transporter | 8.04683453 | 8.1438517 | 8.09335468 | 11.6071769 | 11.5708966 | 11.5501014 | -3.483428 | 0 | 14 |
| CNAG_05387 |  | galactose transporter | 14.1205797 | 14.1073258 | 14.1328441 | 17.5743719 | 17.6161238 | 17.5766562 | -3.4689114 | 0 | 14 |
| CNAG_06493 |  | hypothetical protein | 8.41761411 | 8.41948614 | 8.34673891 | 11.8427215 | 11.8574082 | 11.8281267 | -3.4462792 | 0 | 13 |
| CNAG_13203 |  | ncRNA gene | 3.84607213 | 3.31096168 | 2.71253289 | 7.00706038 | 7.23082115 | 7.47892564 | -3.8385407 | 1.2085E-31 | 14 |
| CNAG_06431 |  | acyl-CoA oxidase | 10.1987287 | 10.314339 | 10.3333636 | 13.5402568 | 13.5558277 | 13.5126008 | -3.2551703 | 0 | 13 |
| CNAG_00679 |  | hypothetical protein | 8.05552549 | 7.9432299 | 8.16648938 | 11.2440247 | 11.4243933 | 11.2407306 | -3.2501147 | 6.29E-279 | 1 |
| CNAG_06628 |  | aldehyde dehydrogenase (NAD) | 11.6480499 | 11.698979 | 11.6710856 | 14.8883629 | 14.9493649 | 14.888187 | -3.236367 | 0 | 7 |
| CNAG_04704 |  | MFS transporter, SHS family, lactate transporter | 2.48350205 | 2.98903359 | 1.12757039 | 6.54515627 | 6.59946575 | 6.20039014 | -4.0503066 | 4.1079E-20 | 10 |
| CNAG_00433 |  | hypothetical protein | 9.02929395 | 9.19360473 | 9.21503324 | 12.3761348 | 12.355924 | 12.3425918 | -3.2143437 | 0 | 1 |
| CNAG_12740 |  | ncRNA gene | 5.06846455 | 5.2369611 | 4.77142658 | 8.13011877 | 8.28459241 | 8.32201958 | -3.2004377 | 6.6801E-57 | 8 |
| CNAG_01954 |  | aldo-keto reductase | 7.9097668 | 7.74392109 | 7.86228001 | 11.0418405 | 11.0401314 | 10.9846614 | -3.1803103 | 5.367E-288 | 11 |
| CNAG_12080 |  | ncRNA gene | 2.48350205 | 1.98903359 | 0.12757039 | 6.26017931 | 5.73439533 | 5.93735573 | -4.096004 | 6.1518E-15 | 1 |
| CNAG_05229 |  | Stomatin family protein | 10.7026706 | 10.6685137 | 10.6821592 | 13.8063642 | 13.8608233 | 13.8943933 | -3.1692292 | 0 | 4 |
| CNAG_00997 |  | hypothetical protein | 7.12735824 | 6.98903359 | 7.47629855 | 10.335889 | 10.3427822 | 10.4595231 | -3.1748947 | 6.485E-175 | 5 |
| CNAG_01384 |  | hypothetical protein | 7.70957013 | 7.8219236 | 7.95411888 | 10.9688866 | 10.9417583 | 11.0285854 | -3.1541967 | 1.297E-266 | 5 |
| CNAG_05666 |  | hypothetical protein | 0 | 0 | 0 | 3.13916391 | 3.833931 | 3.61542764 | -6.066134 | 1.17E-05 | 14 |
| CNAG_05990 |  | hypothetical protein | 8.25734771 | 8.17390893 | 7.94774936 | 11.2202478 | 11.318284 | 11.178518 | -3.1018327 | 4.288E-262 | 12 |
| CNAG_04096 |  | racemase | 8.82080537 | 8.73050057 | 8.74962221 | 11.8012982 | 11.8218582 | 11.7527034 | -3.0232043 | 0 | 2 |
| CNAG_03650 |  | hypothetical protein | 8.28717193 | 8.15394051 | 8.35157207 | 11.3045734 | 11.3123636 | 11.2447843 | -3.0223495 | 1.091E-299 | 2 |

Note:

ITR4_GI: The itr4∆ mutant cultured in medium containing both glucose and inositol

WT_GI: The wild type KN99 cultured in medium containing both glucose and inositol
