# Supplementary material for: An inositol receptor Itr4 coordinates carbon catabolite regulation and virulence in Cryptococcus neoformans
Source: PLoS Biol. 2026 Oct 13;24(10):e3003709. doi: 10.1371/journal.pbio.3003709 (PMC13686456; doi:10.1371/journal.pbio.3003709)
Supplement: Supplementary file 6 [file pbio.3003709.s005.docx]

S5 Table. Top 50 genes up-regulated in the *itr4∆* mutant cells

| **Gene ID** |  | **Name** | **Function description** | **ITR4_GI1** | **ITR4_GI2** | **ITR4_GI3** | **WT_GI1** | **WT_GI2** | **WT_GI3** | **log2FoldChange** | **pvalue** | **gene_chr** |
| --- | --- | --- | --- | --- | --- | --- | --- | --- | --- | --- | --- | --- |
| CNAG_05853 |  |  | hypothetical protein | 9.52320447 | 9.67553411 | 9.95252913 | 6.81723582 | 6.62748012 | 6.20039014 | 3.13229996 | 1.6393E-95 | 7 |
| CNAG_03906 |  |  | hypothetical protein | 9.2592299 | 9.29281433 | 9.27222864 | 6.54515627 | 6.19382694 | 5.96906459 | 2.99468108 | 2.1947E-94 | 2 |
| CNAG_05863 |  |  | hypothetical protein | 10.6605656 | 10.5522299 | 10.7884577 | 7.62459074 | 8.01450325 | 7.93735573 | 2.8076444 | 3.877E-151 | 7 |
| CNAG_03539 |  |  | hypothetical protein | 11.0281906 | 11.0107076 | 11.0231457 | 8.37565653 | 8.43086614 | 8.13200316 | 2.69144229 | 9.252E-214 | 8 |
| CNAG_01907 |  | PLK1 | PLK/PLK1 protein kinase | 10.6127851 | 10.4858874 | 10.7237602 | 7.85615481 | 7.99850277 | 7.88844613 | 2.69378035 | 1.222E-161 | 11 |
| CNAG_02771 |  |  | DNA repair and recombination protein RAD54B | 11.2662663 | 11.2118285 | 11.258141 | 8.55532808 | 8.67523325 | 8.52231823 | 2.65671745 | 4.337E-249 | 3 |
| CNAG_00236 |  |  | 8-Amino-7-oxononanoate synthase | 7.69295542 | 7.86555053 | 7.8824579 | 5.10263804 | 5.28459241 | 5.14594235 | 2.63282625 | 6.6132E-36 | 1 |
| CNAG_06487 |  | CHS6 | Chitin synthase | 12.0929447 | 12.0584834 | 12.0810394 | 9.57045257 | 9.54542591 | 9.47616977 | 2.54345899 | 0 | 13 |
| CNAG_06832 |  | KRE62 | glucosidase, variant 1, glucosidase, variant 2 | 9.52163718 | 9.58335819 | 9.69552647 | 7.3010516 | 6.88123671 | 6.98466144 | 2.52550861 | 6.4016E-88 | 5 |
| CNAG_03453 |  | KIP1 | kinesin family member 11 | 8.85107281 | 8.90192292 | 8.73119674 | 6.48966116 | 6.26689041 | 5.80305464 | 2.58852758 | 6.2348E-59 | 8 |
| CNAG_07856 |  | BWC3 | Arylsulfotransferase, putative | 11.7849982 | 11.8906547 | 11.9529267 | 9.42086216 | 9.35749296 | 9.3403204 | 2.50201856 | 2.336E-258 | 10 |
| CNAG_03148 |  |  | nuclear condensin complex protein | 10.9678934 | 10.9004256 | 10.7866744 | 8.20094011 | 8.45060236 | 8.50619857 | 2.50358772 | 1.721E-155 | 8 |
| CNAG_01959 |  |  | Condensin complex subunit 1 | 10.4851113 | 10.5445814 | 10.3149225 | 8.03155494 | 7.99850277 | 7.92123606 | 2.463783 | 3.543E-136 | 11 |
| CNAG_06384 |  |  | DNA repair protein RAD50 | 10.9499427 | 10.8423431 | 10.867351 | 8.40219832 | 8.62748012 | 8.49535118 | 2.3768788 | 2.07E-166 | 13 |
| CNAG_04018 |  |  | hypothetical protein | 12.584915 | 12.6541474 | 12.4579271 | 10.1747878 | 10.2878875 | 10.1852832 | 2.35061047 | 1.497E-250 | 2 |
| CNAG_00680 |  |  | kinetochore protein Nuf2 | 10.2601701 | 10.3311083 | 10.3431034 | 8.02668918 | 8.19850343 | 7.74012338 | 2.29810885 | 1.263E-108 | 1 |
| CNAG_01389 |  |  | hypothetical protein | 10.1599629 | 10.1652067 | 9.98087995 | 7.86708437 | 7.75993042 | 7.78535264 | 2.29895452 | 1.426E-109 | 5 |
| CNAG_03125 |  |  | hypothetical protein | 6.60688447 | 6.93154809 | 6.84181591 | 4.77143213 | 4.31935783 | 4.14594235 | 2.33544559 | 2.888E-16 | 8 |
| CNAG_03239 |  |  | hypothetical protein | 9.27977347 | 9.20820211 | 9.53058242 | 6.94651884 | 7.22166115 | 7.04541548 | 2.26864897 | 1.3271E-63 | 8 |
| CNAG_06324 |  |  | zinc finger protein | 8.11077536 | 7.80921255 | 7.92847029 | 5.48966116 | 6.03556486 | 5.61542764 | 2.220096 | 3.0008E-29 | 13 |
| CNAG_04692 |  |  | thymidylate synthase | 11.0587712 | 11.0758333 | 11.2574973 | 9.01935964 | 9.04078257 | 8.70289048 | 2.19536026 | 6.004E-122 | 10 |
| CNAG_05565 |  |  | hypothetical protein | 6.7950882 | 7.08706567 | 6.51988782 | 3.62459074 | 4.57089659 | 4.53296547 | 2.54244672 | 1.6399E-16 | 14 |
| CNAG_03184 | BUB1 |  | BUB protein kinase | 11.0397344 | 11.1539405 | 10.8965817 | 8.87522754 | 8.81882411 | 8.83351992 | 2.1909922 | 1.777E-139 | 8 |
| CNAG_01539 |  |  | Myo-inositol-1-phosphate synthase | 14.3348049 | 14.2871678 | 14.4821642 | 12.2006701 | 12.2115754 | 12.1481082 | 2.18286926 | 2.191E-280 | 11 |
| CNAG_02051 |  |  | kinesin family member 20/23 | 10.4915304 | 10.6247517 | 10.3539826 | 8.29296925 | 8.43086614 | 8.20039014 | 2.1787861 | 3.167E-105 | 6 |
| CNAG_06162 |  |  | hypothetical protein | 10.519283 | 10.5213895 | 10.3394587 | 8.39466465 | 8.04078257 | 8.40550457 | 2.17779616 | 8.0181E-97 | 12 |
| CNAG_06697 | MPS1 |  | TTK protein kinase | 10.1076447 | 10.2893861 | 10.0208719 | 8.0604098 | 7.96595939 | 7.89671375 | 2.16306688 | 2.9808E-93 | 7 |
| CNAG_02675 | HSL101 |  | CAMK/CAMKL/GIN4 protein kinase | 12.2690713 | 12.3713874 | 12.108067 | 10.1526262 | 10.0250725 | 10.0821273 | 2.16582709 | 1.495E-175 | 3 |
| CNAG_02721 |  |  | leucine repeat containing protein | 10.2230416 | 10.3586309 | 10.1134123 | 8.14366531 | 7.9549464 | 8.13200316 | 2.15823804 | 5.2843E-98 | 3 |
| CNAG_00172 |  |  | kinesin | 9.32719882 | 9.35754005 | 9.34915752 | 7.03640434 | 7.25795728 | 7.2656816 | 2.16085523 | 5.6171E-72 | 1 |
| CNAG_05177 |  |  | DNA polymerase kappa subunit | 10.4484617 | 10.4184403 | 10.4083412 | 8.13464843 | 8.23992336 | 8.43985607 | 2.1591826 | 2.83E-118 | 4 |
| CNAG_00110 | HPI3 |  | hypothetical protein | 9.83475682 | 9.83765653 | 9.77322883 | 7.86162994 | 7.5780921 | 7.66467115 | 2.10519537 | 1.2001E-85 | 1 |
| CNAG_03500 |  |  | osmosensor | 6.57096489 | 6.61839021 | 6.10485032 | 4.34079777 | 3.9270404 | 3.03046513 | 2.53768231 | 1.7543E-13 | 8 |
| CNAG_06567 |  |  | hypothetical protein | 9.640488 | 9.79960522 | 9.55593057 | 7.53833501 | 7.65496086 | 7.57478565 | 2.07873789 | 1.7794E-74 | 7 |
| CNAG_05759 |  |  | acetyl-CoA carboxylase/biotin carboxylase | 13.8168172 | 13.9019229 | 13.6252975 | 11.7431611 | 11.6266129 | 11.7881329 | 2.0658157 | 1.03E-174 | 7 |
| CNAG_03068 |  |  | hypothetical protein | 10.3343586 | 10.2836543 | 10.4083412 | 8.38328986 | 8.20781117 | 8.2656816 | 2.05287327 | 3.403E-109 | 3 |
| CNAG_03044 |  |  | hypothetical protein | 9.20166794 | 9.40899376 | 9.66090013 | 6.55870281 | 7.3699839 | 7.38801714 | 2.28700006 | 1.231E-15 | 3 |
| CNAG_06141 | DUT101 |  | dUTP pyrophosphatase | 9.68036759 | 9.67553411 | 10.0071536 | 7.81158926 | 7.80968345 | 7.61542764 | 2.03921718 | 7.0686E-64 | 12 |
| CNAG_00237 |  |  | 3-isopropylmalate dehydratase | 9.87827095 | 10.0725129 | 9.74779022 | 7.83960363 | 7.81578365 | 8.00009149 | 2.02487297 | 2.4022E-71 | 1 |
| CNAG_07415 |  |  | hypothetical protein | 11.7249745 | 11.8131968 | 11.6618733 | 9.80307776 | 9.71331003 | 9.68867662 | 1.99677273 | 5.149E-174 | 5 |
| CNAG_02544 |  |  | DNA repair protein Swi5/Sae3 | 5.63034344 | 5.79638851 | 6.0817667 | 3.90469866 | 3.38647202 | 4.03046513 | 2.05897677 | 3.3006E-08 | 6 |
| CNAG_00423 |  |  | kinetochore protein Spc24 | 9.82716367 | 9.81558207 | 10.0627354 | 7.74204832 | 8.08185851 | 7.90493425 | 1.9897487 | 3.3912E-67 | 1 |
| CNAG_01572 | CDC25 |  | tyrosine phosphatase | 9.83349406 | 9.87014755 | 9.85378855 | 7.80592051 | 7.91572509 | 7.87176739 | 1.98825782 | 4.4985E-88 | 11 |
| CNAG_01566 |  |  | gamma-tubulin complex component 3 | 9.40066098 | 9.35316824 | 9.24391436 | 7.34079777 | 7.56366502 | 6.90493425 | 2.02247738 | 2.2134E-50 | 11 |
| CNAG_06687 |  |  | hypothetical protein | 11.4091624 | 11.5252808 | 11.0367134 | 9.42641456 | 9.29117506 | 9.36138201 | 1.97806602 | 3.7599E-78 | 7 |
| CNAG_03716 | BLP6 |  | pr4/barwin domain protein | 9.1170163 | 9.26049661 | 9.13599902 | 7.29296925 | 7.3107447 | 7.00009149 | 1.95156549 | 1.4486E-53 | 2 |
| CNAG_03977 |  |  | hypothetical protein | 10.498718 | 10.5145544 | 10.4807172 | 8.58210741 | 8.4188935 | 8.63037798 | 1.95566678 | 4.664E-112 | 2 |
| CNAG_07635 |  |  | kinetochore protein NDC80 | 10.9980752 | 10.9212483 | 11.0531248 | 9.048457 | 9.04598148 | 9.03421727 | 1.94823597 | 2.214E-140 | 6 |
| CNAG_01909 |  |  | signal transducer | 6.34857247 | 6.41529834 | 5.96046041 | 4.40219832 | 4.45060236 | 4.03046513 | 1.93442346 | 1.2394E-09 | 11 |
| CNAG_07817 |  |  | centromeric protein E | 10.5129865 | 10.6019021 | 10.1261608 | 8.55532808 | 8.49309444 | 8.39386987 | 1.94359372 | 8.5045E-63 | 4 |

Note:

ITR4_GI: the itr4∆ mutant cultured in medium containing both glucose and inositol

WT_GI: The wild type KN99 cultured in medium containing both glucose and inositol
