# Supplementary material for: An inositol receptor Itr4 coordinates carbon catabolite regulation and virulence in Cryptococcus neoformans
Source: PLoS Biol. 2026 Oct 13;24(10):e3003709. doi: 10.1371/journal.pbio.3003709 (PMC13686456; doi:10.1371/journal.pbio.3003709)
Supplement: Supplementary file 7 [file pbio.3003709.s006.docx]

S6 Table. Differentially expressed genes involved in glucose metabolism

| **Gene_id** | **Name** | **Functional description** | **ITR4_GI1** | **ITR4_GI2** | **ITR4_GI3** | **WT_GI1** | **WT_GI2** | **WT_GI3** | **log2FoldChange** | **pvalue** | **gene_chr** |
| --- | --- | --- | --- | --- | --- | --- | --- | --- | --- | --- | --- |
|  | **Glucose transporter** | |  |  |  |  |  |  |  |  |  |
| CNAG_03772 | HXS1 | high-affinity glucose transporter | 7.9097668 | 7.96631351 | 8.01021344 | 15.2037046 | 15.2215176 | 15.1451293 | -7.231482639 | 0 | 2 |
| CNAG_03438 | HXT1 | hexose transporter | 16.3427179 | 16.3411632 | 16.4188131 | 15.904077 | 15.9285586 | 15.8978404 | 0.457762077 | 1.41E-22 | 8 |
| CNAG_04931 | HXS2 | Putative hexose transporter | 1.67614713 | -0.0109664 | #NUM! | 1.40219832 | #NUM! | #NUM! | 0.622678075 | 0.75968774 | 10 |
| CNAG_01862 |  | hexose transporter | 6.88560049 | 6.64724507 | 6.79999574 | 9.17258692 | 9.23082115 | 9.25285756 | -2.43298623 | 7.74E-80 | 11 |
| CNAG_07641 |  | Monosaccharide transporter | 6.83601846 | 6.57399609 | 6.8824579 | 8.87793175 | 8.88993641 | 8.88429449 | -2.113005494 | 2.34E-54 | 6 |
| CNAG_05324 |  | Sugar transporter | 4.63034344 | 2.98903359 | 4.51988782 | 6.30908892 | 6.48162926 | 5.93735573 | -2.042883469 | 7.25E-09 | 4 |
| CNAG_02254 | LPI12 | quinate permease? | 7.34148304 | 7.66145893 | 7.56219862 | 9.49671592 | 9.5120029 | 9.3403204 | -1.932467853 | 1.04E-59 | 6 |
| CNAG_01936 |  | Sugar transporter | 6.43103463 | 6.07649643 | 6.71253289 | 8.18355803 | 8.15585909 | 8.29725167 | -1.785778098 | 6.46E-26 | 11 |
| CNAG_01759 |  | vacuolar membrane protein? | 8.08553806 | 8.0114014 | 8.32232725 | 9.91395097 | 9.88993641 | 9.76743073 | -1.71853684 | 1.21E-60 | 11 |
| CNAG_04092 |  | Sugar transporter | 8.01153748 | 7.99465814 | 8.18285283 | 9.71356822 | 9.67187424 | 9.52498742 | -1.579474729 | 1.76E-50 | 2 |
| CNAG_02586 |  | Sugar transporter | 6.44433145 | 6.5281924 | 6.79999574 | 8.22237728 | 8.06146672 | 8.04541548 | -1.523471642 | 5.91E-21 | 3 |
| CNAG_05929 |  | General alpha glucoside:H symporter | 8.82335205 | 8.88687904 | 8.83147397 | 10.168175 | 10.1938269 | 10.0748593 | -1.3012528 | 3.28E-52 | 7 |
| CNAG_06253 |  | Sugar transporter | 6.85605622 | 6.91977092 | 6.92198626 | 8.11644384 | 8.18913525 | 7.90493425 | -1.182524245 | 1.61E-14 | 12 |
| CNAG_06527 |  | General alpha glucoside:H symporter, | 6.19970908 | 6.32888359 | 6.2974954 | 7.43928764 | 7.62052736 | 7.13200316 | -1.145253157 | 4.52E-09 | 7 |
| CNAG_01055 |  | Phospholipid transporter, variant | 13.0324615 | 12.9702441 | 13.1803082 | 14.2130377 | 14.2095497 | 14.1567394 | -1.129990545 | 4.27E-77 | 5 |
| CNAG_00048 |  | Sugar transporter | 6.21530594 | 5.87167664 | 5.79999574 | 7.01690816 | 6.94940822 | 7.06021248 | -1.023728467 | 1.36E-06 | 1 |
| CNAG_01059 |  | Vacuolar membrane protein, variant | 10.9487769 | 10.9548179 | 11.1041345 | 12.0030404 | 11.9597749 | 12.0148836 | -0.989257943 | 8.87E-55 | 5 |
| CNAG_03087 |  | Maltose permease | 5.58303772 | 6.32888359 | 5.71253289 | 6.55870281 | 6.96046341 | 6.95329727 | -0.923107893 | 0.00016358 | 8 |
| CNAG_04210 |  | General alpha glucoside:H symporter | 11.6779072 | 11.651702 | 11.707829 | 12.4525005 | 12.5253256 | 12.5326339 | -0.824334613 | 6.05E-51 | 9 |
| CNAG_04038 |  | MFS quinate transporter QutD | 8.72599568 | 8.71354744 | 8.77142658 | 9.47723171 | 9.57269884 | 9.58249375 | -0.806837328 | 4.79E-18 | 2 |
| CNAG_06259 |  | General alpha glucoside:H symporter | 9.64337339 | 9.67553411 | 9.72002743 | 10.4619934 | 10.5176275 | 10.4170462 | -0.788372702 | 7.34E-26 | 13 |
| CNAG_07874 |  | Sugar transporter | 0.67614713 | -0.0109664 | 3.12757039 | 2.98716082 | 2.73439533 | 2.03046513 | -0.78510939 | 0.41164969 | 14 |
| CNAG_06521 |  | hypothetical protein | 8.43769836 | 8.50866984 | 8.75327924 | 9.28076019 | 9.41085618 | 9.32814568 | -0.772375616 | 8.65E-13 | 13 |
| CNAG_01835 |  | hypothetical protein | 12.5032913 | 12.4657798 | 12.5353692 | 13.2386432 | 13.2738311 | 13.1867589 | -0.732188962 | 1.15E-44 | 11 |
| CNAG_06292 |  | Sugar transporter, variant 2 | 7.74760949 | 7.98338702 | 7.65113235 | 8.47187185 | 8.39871562 | 8.37623997 | -0.616599619 | 2.64E-06 | 13 |
| CNAG_03140 |  | Sugar transporter | 15.0014526 | 15.0260228 | 15.1336334 | 15.4614019 | 15.4927375 | 15.4022871 | -0.397933723 | 1.55E-13 | 8 |
| CNAG_05336 |  | Glucose transporter, variant 2 | 11.6775553 | 11.7007006 | 11.6879032 | 12.0469559 | 12.0345844 | 11.9328402 | -0.318111161 | 3.26E-08 | 14 |
| CNAG_06934 |  | protein, variant 3 | 10.3843689 | 10.3553558 | 10.4539999 | 10.7006428 | 10.5527492 | 10.4734086 | -0.183162191 | 0.01826898 | 3 |
| CNAG_02554 |  | Sugar transporter | 11.230736 | 11.1905449 | 11.3303064 | 11.3646124 | 11.3129028 | 11.2720511 | -0.066765707 | 0.29949813 | 6 |
| CNAG_06986 | LPI8 | Sugar transporter | 9.99129669 | 10.0072338 | 9.94774936 | 10.0045879 | 9.94802034 | 10.1442073 | -0.048749867 | 0.54344868 | 12 |
| CNAG_04920 |  | Galactose transporter, variant | 12.2556988 | 12.2695144 | 12.2500749 | 12.1690033 | 12.1516451 | 12.1014805 | 0.116818441 | 0.02617804 | 10 |
| CNAG_06485 |  | Hexose transporter protein | 9.2441032 | 9.18625028 | 9.52844983 | 9.14366531 | 9.21244262 | 8.96906459 | 0.209568833 | 0.05845734 | 13 |
| CNAG_02479 |  | Monosaccharide transporter | 7.85605622 | 7.89592418 | 7.84866958 | 7.30908892 | 7.19382694 | 7.10371412 | 0.656478387 | 1.06E-05 | 6 |
| CNAG_05459 |  | pi-transporter A-1 | 10.4057679 | 10.4788815 | 10.3260154 | 9.81017416 | 9.7850214 | 9.61292078 | 0.662658089 | 8.49E-16 | 14 |
| CNAG_05914 |  | General alpha glucoside:H symporter | 8.47380865 | 8.48088668 | 8.64720665 | 7.67521681 | 8.13161155 | 7.75838559 | 0.662925448 | 2.11E-06 | 7 |
| CNAG_06963 |  | Sugar transporter | 11.5905323 | 11.5402612 | 11.5559306 | 10.9192115 | 10.9028884 | 10.8717674 | 0.66380492 | 3.76E-29 | 3 |
| CNAG_05330 |  | General alpha glucoside:H symporter | 5.76360997 | 5.66145893 | 6.14993821 | 5.24350057 | 4.833931 | 5.44550263 | 0.685904182 | 0.02300268 | 4 |
| CNAG_04783 |  | Monosaccharide transporter | 11.4133945 | 11.3504291 | 11.5123541 | 10.4816831 | 10.5500073 | 10.4539313 | 0.929753064 | 8.90E-42 | 10 |
| CNAG_04784 |  | Monosaccharide transporter | 3.67614713 | 3.89592418 | 3.2974954 | 1.81723582 | 2.73439533 | 3.25285756 | 0.975826836 | 0.13824303 | 10 |
| CNAG_01683 | STL1 | Monosaccharide transporter | 11.9505253 | 11.9562598 | 11.9979351 | 10.7959463 | 10.8043245 | 10.7251132 | 1.191118423 | 2.19E-90 | 11 |
|  | **Glycolysis** |  |  |  |  |  |  |  |  |  |  |
| CNAG_05480 | HXK1 | Phosphorylates glucose to glucose-6-phosphate; | 13.6184784 | 13.5796206 | 13.704409 | 13.5136601 | 13.5357573 | 13.4638767 | 0.130006291 | 0.01525152 | 14 |
| CNAG_03769 | HXK2 | Hexokinase; glucose sensing/repression | 14.792816 | 14.7806544 | 14.8886021 | 14.9005074 | 14.9197133 | 14.8713086 | -0.075951458 | 0.13131921 | 2 |
| CNAG_04676 | PFK1 | Phosphorylates fructose-6-p to fructose-1,6-bisp | 11.5766366 | 11.6525917 | 11.2943611 | 12.5306223 | 12.5169256 | 12.5847342 | -1.027461635 | 3.25E-38 | 10 |
| CNAG_01820 | PYK1 | Converts phosphoenolpyruvate to pyruvate | 14.0083935 | 14.0335985 | 13.9797097 | 13.5198414 | 13.5242782 | 13.6143314 | 0.45436603 | 3.42E-19 | 11 |
|  | **Gluconeogenesis** |  |  |  |  |  |  |  |  |  |  |
| CNAG_00057 | FPP1 | Dephosphorylates fructose-1,6-bisp to fructose-6-p | 14.5081965 | 14.4343718 | 14.6080451 | 13.9515819 | 13.9645016 | 13.9330915 | 0.568711769 | 1.55E-24 | 1 |
| CNAG_04217 | PCK1 | Converts oxaloacetate to phosphoenolpyruvate; | 12.6370541 | 12.6213472 | 12.6179199 | 14.8000957 | 14.796827 | 14.8926504 | -2.204816458 | 0 | 9 |
|  | **Carbon Catabolite Repression (CCR) Regulators** | |  |  |  |  |  |  |  |  |  |
| CNAG_06552 | SNF1 | AMPK ortholog | 10.6339747 | 10.7237432 | 10.5961944 | 10.2009401 | 10.1437863 | 10.2688699 | 0.448795639 | 8.10E-10 | 7 |
| CNAG_06327 | MIG1 | Glucose repressor | 11.8086469 | 11.8838514 | 11.7532792 | 12.5366246 | 12.5495499 | 12.5478065 | -0.728189131 | 1.17E-38 | 13 |
|  | **Inositol transporter gene family** | |  |  |  |  |  |  |  |  |  |
| CNAG_05662 | ITR4 | myo-inositol transceptor | 0 | 0 | 0 | 14.6911605 | 14.6695604 | 14.6682221 | -17.18813268 | 3.88E-48 | 14 |
| CNAG_05667 | ITR3B | myo-inositol transporter | 0 | 0 |  | 10.2787153 | 10.4248922 | 10.320484 | -12.85462538 | 1.39E-27 | 14 |
| CNAG_05381 | ITR3C | myo-inositol transporter, putative | 6.39039265 | 6.95481787 | 7.02238816 | 9.62459074 | 9.65155426 | 9.58505399 | -2.822871077 | 1.56E-96 | 14 |
| CNAG_03910 | ITR6 | myo-inositol transporter, putative | 11.0148835 | 10.9410468 | 11.2491039 | 12.8691245 | 12.8881283 | 12.842375 | -1.793510729 | 5.08E-132 | 2 |
| CNAG_00864 | ITR2 | myo-inositol transporter, putative | 7.24600274 | 7.15895859 | 7.44949849 | 9.63101701 | 9.5600356 | 9.85913056 | -2.399394481 | 3.84E-84 | 5 |
| CNAG_00097 | ITR1 | myo-inositol transporter, putative | 10.2211116 | 10.2848025 | 10.1275704 | 11.3875659 | 11.3438379 | 11.2807636 | -1.125745707 | 2.65E-59 | 1 |
| CNAG_00867 | ITR3A | myo-inositol transporter, putative | 8.11493898 | 8.22743833 | 7.94774936 | 9.27871527 | 9.16066008 | 9.03046513 | -1.060794147 | 9.26E-20 | 5 |
| CNAG_04024 | ITR5 | myo-inositol transporter | 8.92169983 | 9.0361575 | 9.09911395 | 9.95550762 | 9.85036789 | 9.83567159 | -0.864955747 | 8.27E-22 | 2 |
| CNAG_04552 | ITR1A | putative myo-inositol transporter | 6.92407464 | 7.15895859 | 7.43135114 | 7.57878705 | 7.29336262 | 7.2656816 | -0.212347097 | 0.23896217 | 10 |
| CNAG_05377 | ITR3 | myo-inositol transporter, putative | 7.97535515 | 8.0923214 | 8.09335468 | 8.02180696 | 8.16066008 | 8.22028969 | -0.079777893 | 0.53311559 | 14 |

**Note:**

ITR4_GI: the itr4∆ mutant cultured in medium containing both glucose and inositol

WT_GI: The wild type KN99 cultured in medium containing both glucose and inositol
