# Supplementary material for: An inositol receptor Itr4 coordinates carbon catabolite regulation and virulence in Cryptococcus neoformans
Source: PLoS Biol. 2026 Oct 13;24(10):e3003709. doi: 10.1371/journal.pbio.3003709 (PMC13686456; doi:10.1371/journal.pbio.3003709)
Supplement: Supplementary file 8 [file pbio.3003709.s007.docx]

S7 Table. SEC-MALS analysis of shed extracellular polysaccharide (EPS) from different strains

| **Samples** | **Peak** | **Mass (µg)** | **MW (kDa)** | **Polydispersity** | **Radius (nm)** |
| --- | --- | --- | --- | --- | --- |
| KN99_Glu | 1 | 181.63 | 9.36 | 1.64 | 52.90 |
|  | 2 | 10.98 | 32.38 | 1.85 | 56.80 |
| KN99_Ino | 1 | 215.31 | 4.54 | 1.45 | 55.00 |
|  | 2 | 9.82 | 23.10 | 1.30 | 80.50 |
| *itr4*∆_Glu | 1 | 176.05 | 4.77 | 1.58 | 72.20 |
|  | 2 | 6.90 | 28.67 | 1.40 | 113.00 |
| *itr4*∆_Ino | 1 | 237.50 | 1.77 | 1.40 | 41.20 |
|  | 2 | 1.94 | 132.10 | 1.01 | 96.50 |
| *ITR4^G388A^ ^Q389A^*_Glu | 1 | 174.81 | 2.72 | 1.41 | 49.80 |
|  | 2 | 2.64 | 29.87 | 1.09 | 63.70 |
| *ITR4^Q388A^ ^Q389A^*_Ino | 1 | 186.67 | 2.44 | 1.55 | 52.50 |
|  | 2 | 34.96 | 3.67 | 1.28 | 87.00 |
